# Optimizing a photon absorber using conformal cooling channels and additive manufacturing in copper

**DOI:** 10.1107/S1600577525003078

**Published:** 2025-05-13

**Authors:** Younes Chahid, Carolyn Atkins, Stephen Hodbod, John Robinson, Xia Liu, Stephen Watson, Maia Jones, Mark Cliffe, Dayo Ogunkanmi, Richard Kotlewski, Lee Chapman, Scott Beamish, Jorge Linde Cerezo, Thomas Wearing, Ahmad Baroutaji, Arun Arjunan, Chantal Fowler, Paul Vivian

**Affiliations:** a UK Astronomy Technology Centre Royal Observatory EdinburghEH9 3HJ United Kingdom; b Diamond Light Source DidcotOX11 0DE United Kingdom; c Additive Manufacturing Functional Materials Research Group University of Wolverhampton TelfordTF2 9NT United Kingdom; d Additive Analytics, Elite Centre for Manufacturing Skills University of Wolverhampton Springfield Campus WolverhamptonWV10 0JP United Kingdom; e School of Physics Engineering and Technology University of York YorkYO10 5DD United Kingdom; f School of Engineering and Innovation Aston University Aston Triangle BirminghamB4 7ET United Kingdom; g ESA-RAL Advanced Manufacturing Laboratory, STFC, Harwell Campus, DidcotOX11 0DE, United Kingdom; Paul Scherrer Institute, Switzerland; EPFL, Switzerland

**Keywords:** additive manufacturing, 3D printing, particle accelerator, heat transfer, pressure drop

## Abstract

Many of the 70 synchrotron facilities worldwide are undergoing upgrades to their infrastructure to meet a growing demand for increased beam brightness with nanometre-level stability. These upgrades increase the mechanical and thermal challenges faced by beamline components, creating opportunities to apply novel methodologies and manufacturing processes to optimize hardware performance and beam accuracy. Absorbers are important beamline components that rely on water-cooled channels to absorb thermal energy from excess light caused by synchrotron radiation or photon beams created by insertion devices, all within a limited volume, to protect downstream equipment and ensure safe, reliable operation. Additive manufacturing (AM) has been shown to meet criteria relevant to synchrotron environments like leak tightness and vacuum compatibility. However, there is a research gap on the heat transfer and pressure drop impact of different AM conformal cooling channel geometries, as well as the print quality of AM copper parts using low-power infrared lasers and their compliance with absorber requirements. In this study, an intermediate model of a Diamond Light Source photon absorber was optimized to incorporate AM conformal cooling channels, leading to two concept designs named ‘Horizontal’ and ‘Coil’. When compared with the baseline design, the lightweight Horizontal concept performed the best in this study, with simulations showing a maximum temperature drop of 11%, a calculated pressure drop reduction of 82%, a mass reduction of 86%, and the consolidation of 21 individually brazed pipes into a single manifold. The AM print quality and compliance with the synchrotron environment was examined by producing custom benchmark artefacts and measuring their surface roughness, dimensional accuracy and porosity levels, which are characteristics that can affect heat absorption, structural integrity, thermal conductivity and vacuum performance. The study demonstrates the benefits and addresses outstanding challenges in reducing thermal fatigue, as well as the size, vibrations and energy consumption of AM absorbers.

## Abstract 2

This study optimizes a Diamond Light Source photon absorber using additive manufactured (AM; 3D printing) conformal cooling channels in copper, achieving a maximum temperature drop of 11%, pressure drop reduction of 82% and examination of the AM print quality and its compliance with synchrotron and particle accelerator hardware applications using custom benchmark artefacts. These improvements can reduce thermal fatigue failure, component size, vibrations and energy consumption of absorbers, boosting overall facility efficiency, reliability and beam stability.

## 1. Introduction

**Figure 1:**
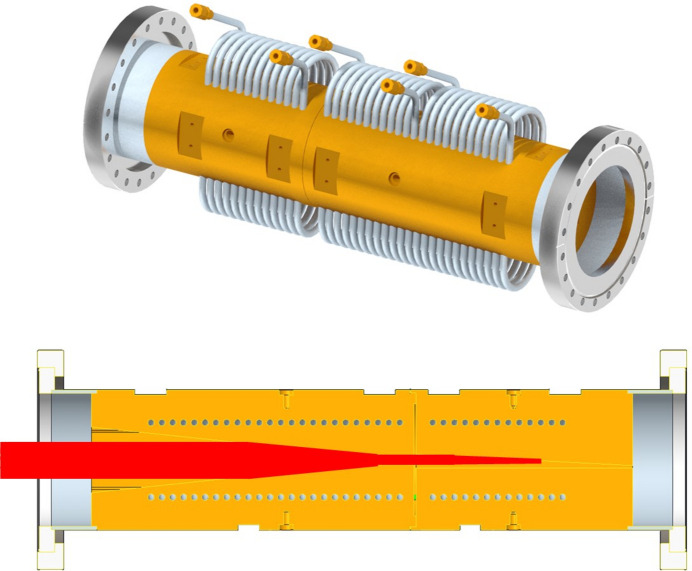
Full size conventional absorber CAD design with a length of 600 mm (top). Cross section of the conventional CAD-design, showing an example of the beam in red, coming from the insertion device, and hitting the BTS which is angled at 3.5° to the source (bottom).

Diamond Light Source (DLS) is the UK’s national synchrotron facility, operating at an energy of 3 GeV and serving more than 9000 scientists annually across 33 beamlines (DLS, 2023 ▸). The synchrotron is equipped with ∼100 absorbers that act as heat sinks, effectively absorbing the thermal energy from excess light caused by synchrotron radiation (SR) or powerful photon beams created by insertion devices. They are crucial for the safe and reliable operation of the synchrotron and are custom designed for different tasks like collimating the insertion device beam or, in the case of a ‘front end’ absorber, blocking it entirely to protect downstream components of the beamline. An example of a front end absorber can be seen in Figs. 1 ▸ and 2 ▸.

The operation of the absorber can be summarized in three main steps. First, the illuminated absorber surface comes into contact with the beam generated by the cryogenic permanent magnet undulator (CPMU) (Sharma *et al.*, 2024 ▸). This interaction causes thermal radiation heat transfer, resulting in a temperature increase of the surface that touches the beam. In the remainder of this study, this surface will be referred to as the beam-touching surface (BTS), and can be seen in Fig. 1 ▸ (bottom). Second, the absorbed heat from the BTS is carried through conductive heat transfer to the surfaces in contact with the cooling channel fluid. Third, the heat from the pipe channel surface is removed by the movement of the fluid, causing a heat dissipation governed by convective heat transfer.

### 1.1. Challenges with conventional absorber design and manufacturing techniques

One of the main challenges in designing an absorber is selecting the optimal BTS angle. A 90° angle results in a BTS that is directly perpendicular to the beam. In contrast, an angle less than 90° distributes the SR beam and reduces the SR beam power density. This helps prevent localized melting or overheating, thereby maintaining system stability and safety. However, a smaller BTS angle, such as the 3.5° used for the conventional design in this study (Fig. 1 ▸, bottom), increases the length of the absorber, thereby enlarging its overall dimensions and footprint along the path of the SR beam. When this length increase is multiplied across the numerous absorbers in the synchrotron, the resulting footprint can become substantial and problematic. This challenge highlights the need for improved heat transfer solutions that can achieve shorter absorbers with steeper BTS angles. Furthermore, another critical reason to improve heat transfer is to reduce thermal fatigue.

**Figure 2:**
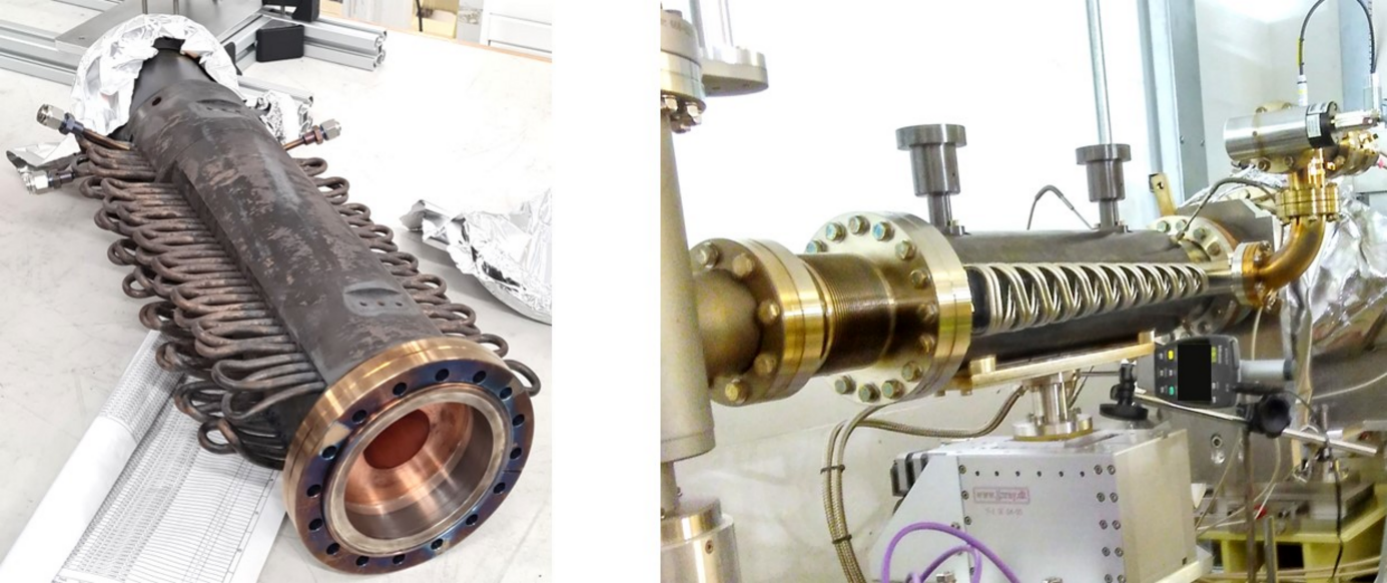
Example of a conventionally manufactured copper absorber showing vacuum brazed copper cooling pipes (left). Assembled version of an absorber in the DLS synchrotron front end with vacuum brazed stainless steel pipes (right).

Conventional techniques in manufacturing an absorber involve wire electrical discharge machining (EDM) of internal profiles creating the BTS angled tapers, and vacuum brazing steel or copper pipes to connect them to the drilled cooling channels, as shown in Fig. 2 ▸. These techniques do not permit the creation of freeform internal geometries, which restricts the design options for cooling channels and the heat transfer performance. Also, the brazing operation and quality assurance required for each individual cooling pipe joint is often the most time-consuming step in the entire manufacturing process. These design and manufacturing challenges highlight the need for alternative techniques, such as additive manufacturing (AM; 3D printing), which can provide greater geometric freedom, allowing for a design approach focused on function, rather than manufacture, leading to improved heat transfer and efficient, shorter absorbers.

### 1.2. AM research

The viability of AM, particularly through the laser powder bed fusion (LPBF) technology, has been explored for particle accelerator applications in different materials including aluminium (Jenzer *et al.*, 2017 ▸; Varnasseri *et al.*, 2021 ▸), stainless steel (Cooper *et al.*, 2021 ▸) and copper (Torims *et al.*, 2021 ▸; Torims *et al.*, 2023 ▸). While previous studies investigated consolidation, lightweighting, leak testing and vacuum compatibility of produced AM components (Romano *et al.*, 2024 ▸; Scott & Omolayo, 2014 ▸), there is a research gap in the use of AM in optimizing the thermal efficiency of absorbers through conformal cooling channels and their impact on heat transfer and pressure drop of the cooling fluid. Optimizing heat transfer through AM conformal channels positively influences length reduction (by increasing BTS angle); lower energy consumption (by using a pipe geometry that lowers fluid pressure drop); and reduced lead time and cost (by consolidating components and reducing the vacuum brazing operations).

During the LPBF process, the high reflectivity of copper in the infrared range can limit laser energy absorption, leading to inadequate melting and part defects. As a result, previous studies have investigated the use of green lasers for the AM of an absorber (Seskauskaite, 2023 ▸) or other accelerator applications such as a radio frequency quadrupole (RFQ) (Torims *et al.*, 2021 ▸; Torims *et al.*, 2023 ▸). However, low-power infrared lasers are the most widely available, and therefore accessible systems within LPBF machines to date (Romano *et al.*, 2024 ▸), and there is a research gap in their use in printing particle accelerator hardware in copper.

In this study, the primary aim was to improve the efficiency of an absorber at the system level by redesigning the cooling channels to enhance heat transfer and reduce the pressure drop of the cooling fluid. Increasing heat transfer allows for shorter absorbers and decreased thermal fatigue. Likewise, a decrease in fluid pressure drop reduces the energy usage and flow rate leading to reduced vibrations that can affect the performance of connected or nearby sensitive equipment, for example X-ray reflecting synchrotron mirrors. To achieve this aim, two types of conformal cooling channels were designed on a shortened DLS absorber (Section 2.1). The concept designs were thermally simulated (Section 2.2) and pressure drop tested using 3D printed resin prototypes (Section 3.1).

The secondary aim was to evaluate the compliance of the low-power infrared AM process with the absorber operational requirements, as LPBF printed components frequently require post-processing. For example, AM parts require post-machining to provide interfaces with suitable dimensional accuracy for mechanical joining. Also, the relatively large surface roughness of as-built AM parts in comparison with machined counterparts may lead to both longer degassing times and increased photon scattering that can cause localized heating of adjacent surfaces. In addition, as built AM porosity cannot only reduce the thermal conductivity of a material but it can provide a leak path and a further source of trapped air molecules. To achieve this aim, benchmark artefacts were designed, 3D printed and inspected (Sections 3.2 to 3.5). This step allowed for the quantification of AM process characteristics and reliable copper printing of the intermediate concept models of the absorber (Section 3.6). Finally, the study evaluates how these results impact full-size absorbers and explores the overall advantages and challenges of using AM for particle accelerator hardware (Section 4).

## 2. Design and thermal simulation

### 2.1. Conventional design and thermal simulation

**Figure 3:**
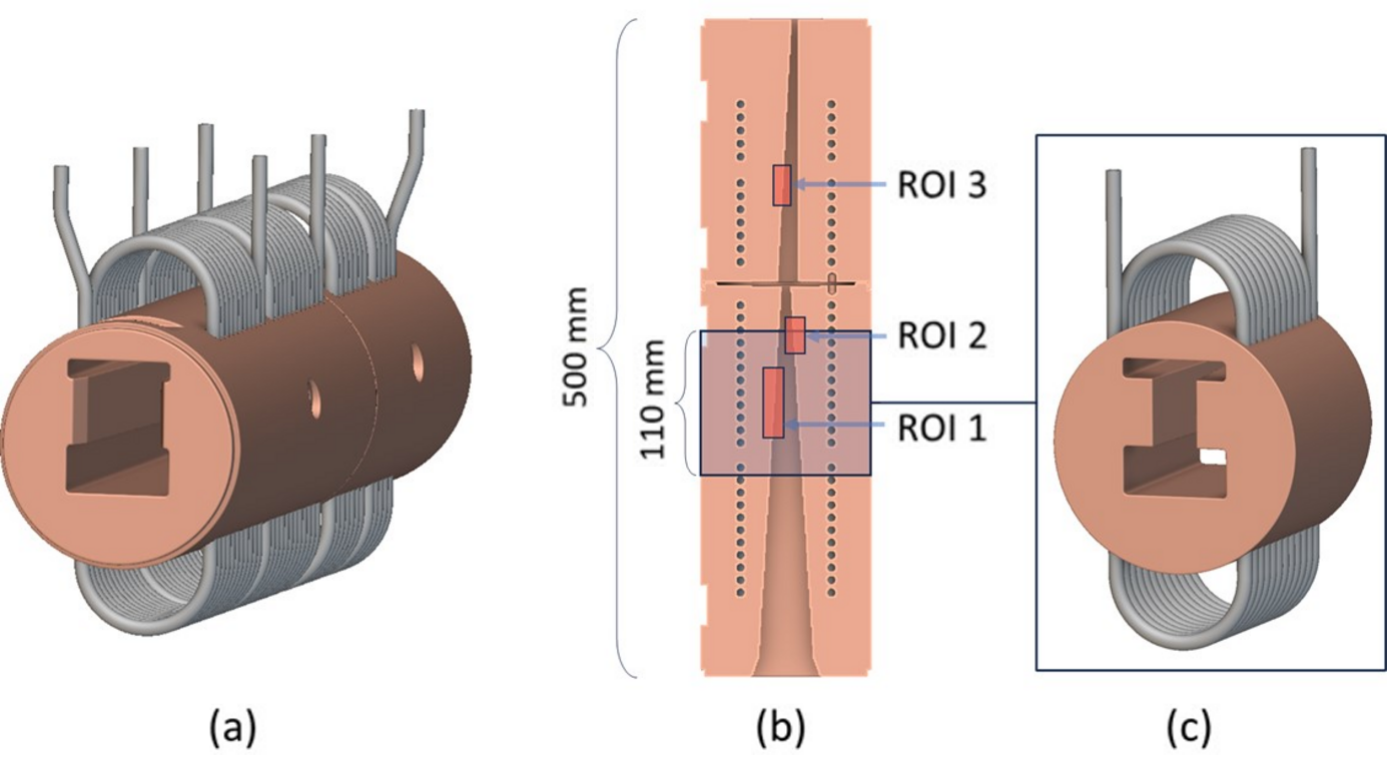
Conventional full size design (*a*), ROIs of different beam configurations, touching different BTS and resulting in different maximum temperatures (*b*), and trimmed design forming the intermediate model used for concept development (*c*).

**Figure 4:**
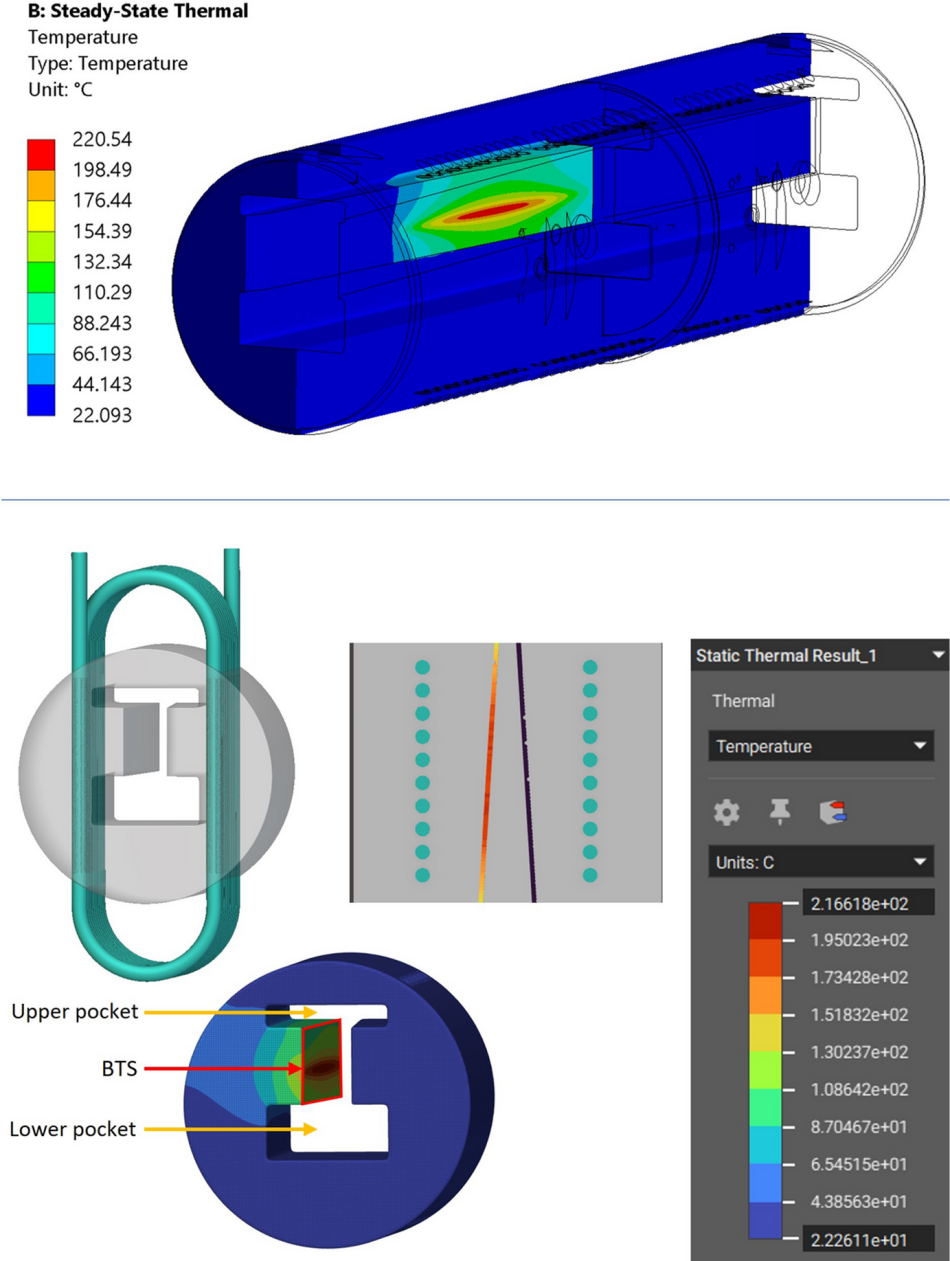
Full size conventional CAD cross section showing the thermal simulation result in *Ansys* (top). Intermediate model thermal simulation result made using *nTop* and cross section showing cooling channels (bottom).

DLS absorbers are manufactured in oxygen-free high conductivity (OFHC) copper and are composed of two large axial copper bodies and cooling pipes. The chosen absorber is subject to three different beam loading configurations that affect three distinct regions of interest (ROIs). The focus of this study was ROI 1 [Fig. 3 ▸(*b*)] as it reaches the highest temperature in the absorber. The result of this focus can be seen in Fig. 3 ▸(*c*), where the original computer-aided design (CAD) was trimmed to a reduced length of 110 mm and de-featured, forming an intermediate design that uses less computing power for meshing/simulation and allows for faster design iterations. To validate the intermediate design, a linear steady state thermal simulation was first performed on the full size CAD model (Fig. 4 ▸, top), using *Ansys Workbench 2020 R2* (Synopsis, USA) and the boundary conditions in Table 1 ▸, resulting in a maximum temperature of 220.5°C. The boundary conditions included the beam profile and gradient heat flux, as well as the cooling convection coefficient and ambient temperature. The heat dissipated by air convection is negligible compared with water convection and therefore out of scope for this study. The water convection coefficient calculation was based on the Prandtl number equation and Dittus-Boelter equation for the Nusselt number [Appendix *A*[App appa], equations (1) and (2)] and can be seen in Appendix *B*[App appb].

The same boundary conditions were applied on the intermediate design (Fig. 4 ▸, bottom), using *nTop 4.18.2* (Ntopology, USA), and with cooling channels that have the same diameter, spacing and distance from the BTS to match the original CAD. The result was a maximum temperature of 218.3°C, which is 1.0% lower than the original CAD simulation maximum temperature; therefore, due to the low variation, the intermediate model was adopted as the baseline/benchmark for the study.

### 2.2. Two AM design concepts and thermal simulation

Two AM design concepts were developed, termed ‘Horizontal’ and ‘Coil’. The primary objective of these designs was to ensure the cooling channels conformed to the BTS and were consolidated within the absorber geometry. Conformal means creating cooling channels that closely follow the geometry of the BTS, increasing the thermal gradient and heat transfer between the two features. Consolidated means integrating the absorber and cooling channels into a single component, which decreases the number of parts in the assembly and reduces manufacturing time and costs associated with the manual brazing of individual pipes (Fig. 2 ▸, right). Furthermore, consolidation also results in a monolithic copper cooling channel with improved thermal conductivity by eliminating the use of steel pipes in the 180° return bends, and removing the thermal resistance from brazed joints (Corbin *et al.*, 2014 ▸).

**Figure 5:**
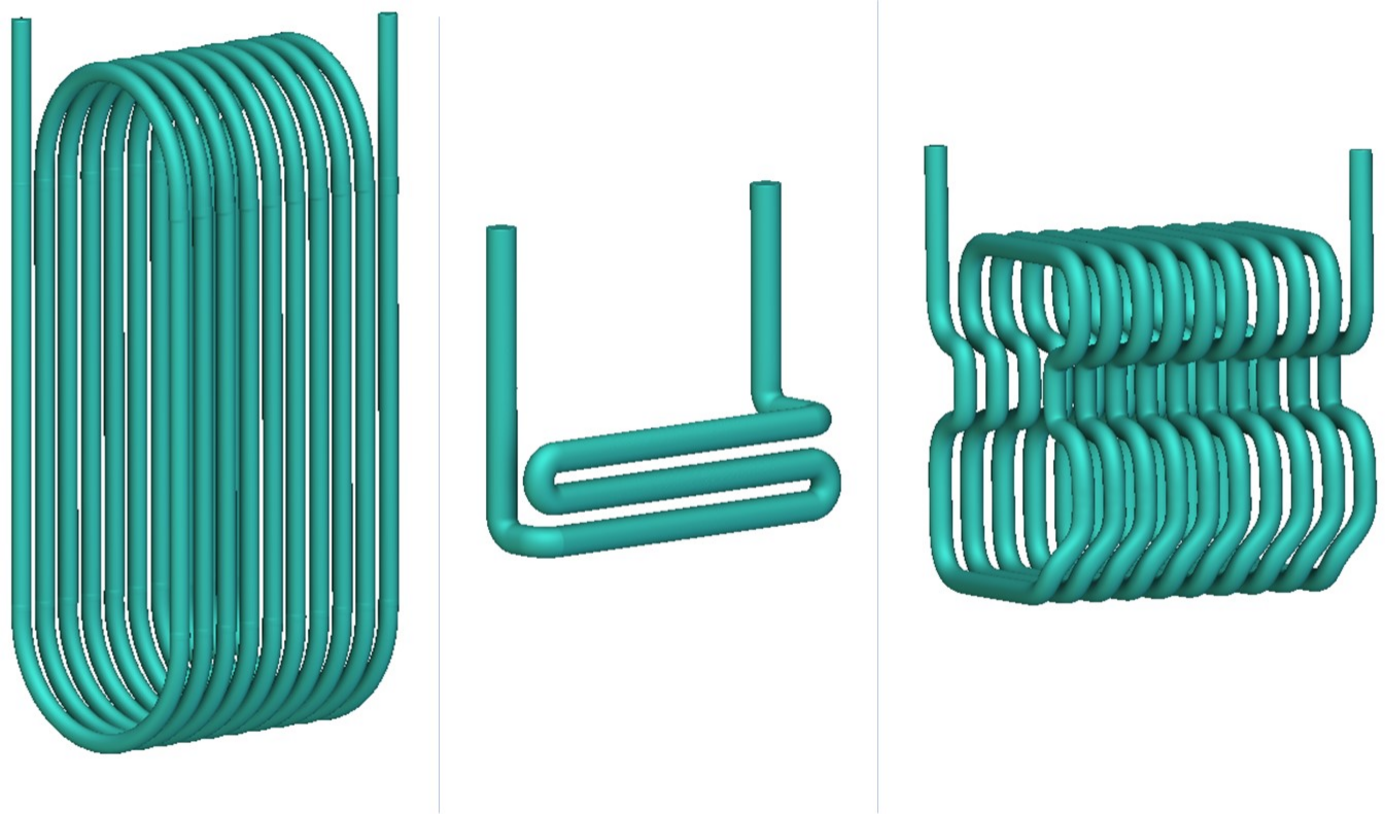
Design of the cooling channels used in the intermediate model (left), in the Horizontal model (middle) and in the Coil model (right).

The Horizontal model (Fig. 5 ▸, middle) was designed in *Inventor2023* (Autodesk, USA) to have a minimum pipe length and number of pipe bends, important factors in reducing pressure drop. The inner pipe was enlarged from the original CAD size of 6 mm-diameter to 8 mm-diameter, to increase the heat transfer surface area. In contrast, the Coil model (Fig. 5 ▸, right) was designed in *Grasshopper Rhino 8* (Robert McNeel & Associates, USA) to maximize the cooling performance of the part using densely populated channels (advantageous for versatile absorbers) and by having a single inlet and outlet for two opposite BTSs, similar to the conventional design.

In the Horizontal design, the cooling channel distance to the BTS is 4.7 mm and the Coil design cooling channel’s distance to the BTS is 5.7 mm. Both of these designs had a closer distance of the cooling channels to the BTS in comparison with the conventional CAD distance of ∼20 mm, as seen in the design cross section of Fig. 4 ▸ (bottom). This increased distance in the conventional CAD is due to two factors: firstly, two pockets above and below the BTS (Fig. 4 ▸, bottom) are needed for EDM machining and for the beam to pass through when blocking is not required, respectively; and secondly, if drilling is done closer to the BTS, it would intersect with these pockets and expose the cooling channel to the vacuum. This challenge explains the motivation to investigate AM for the design of freeform conformal cooling pipes, which enables shorter distances to the BTS while maintaining a safe distance of ≥4 mm to the inner vacuum.

**Figure 6:**
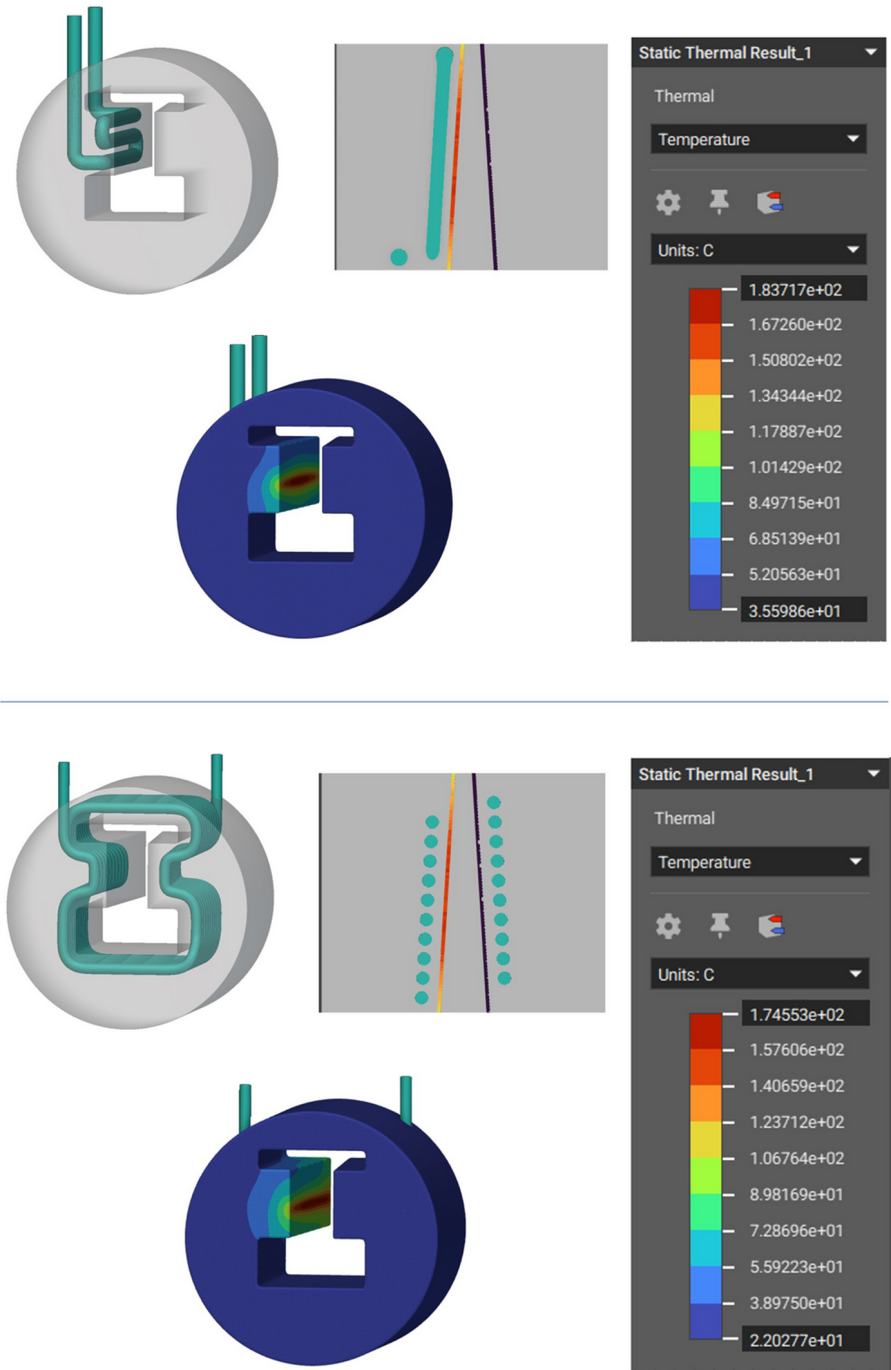
Horizontal model steady state thermal simulation and cross section (top). Coil model steady state thermal simulation and cross section (bottom).

**Figure 7:**
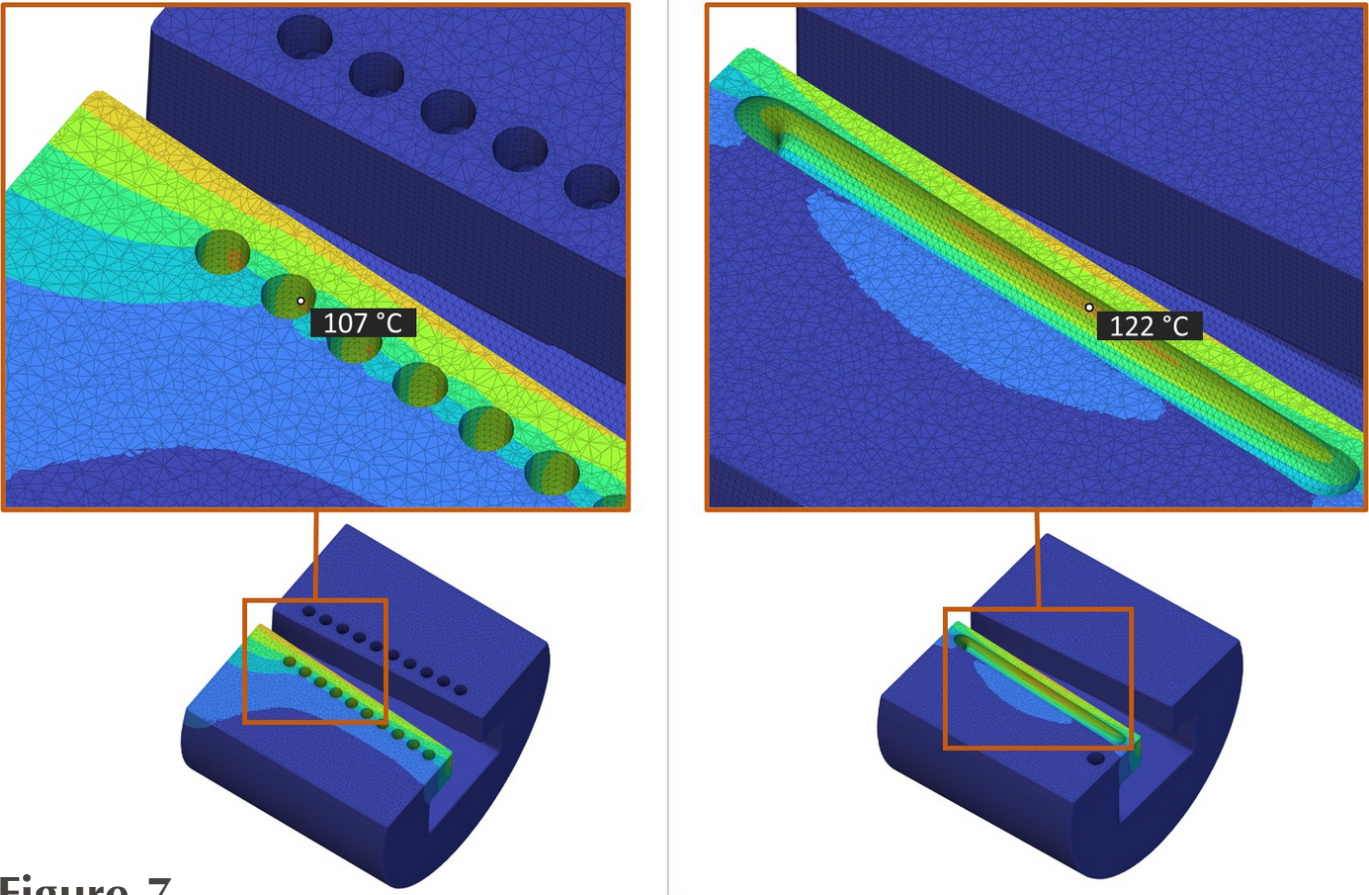
Cooling water interface maximum temperature of ∼107°C for the Horizontal design (left) and ∼122°C for the Coil design. Both temperatures are below the maximum boiling temperature of ∼165°C for DLS synchrotron water operating at 6 bar (gauge pressure).

Linear steady-state thermal simulation was applied on the two generated Horizontal and Coil design concepts using *nTop* and based on the boundary conditions in Table 1 ▸. The resulting maximum temperatures of the Horizontal and Coil cooling channel design were 183.7°C and 174.6°C, respectively (Fig. 6 ▸), showing a respective 15% and 20% lower maximum temperature than the intermediate conventional model simulation (Fig. 4 ▸, bottom), summarized in Table 5. The water cooling convection coefficient of the two concept designs can also be seen in Appendix *B*[App appb]. Furthermore, the maximum cooling water interface temperatures are approximately 107°C for the Horizontal design (Fig. 7 ▸, left) and 122°C (Fig. 7 ▸, right) for the Coil design. Both temperatures are below the maximum boiling point of ∼165°C for water operating at 6 bar (gauge pressure) (NIST, 2023 ▸) which is the standard for the DLS synchrotron. Keeping the water temperature below this boiling point avoids a phase change from liquid to vapour, which is undesirable for absorber operation because it can potentially cause operational issues or damage.

## 3. Additive manufacturing and metrology

### 3.1. Pressure drop calculation and measurement

**Figure 8:**
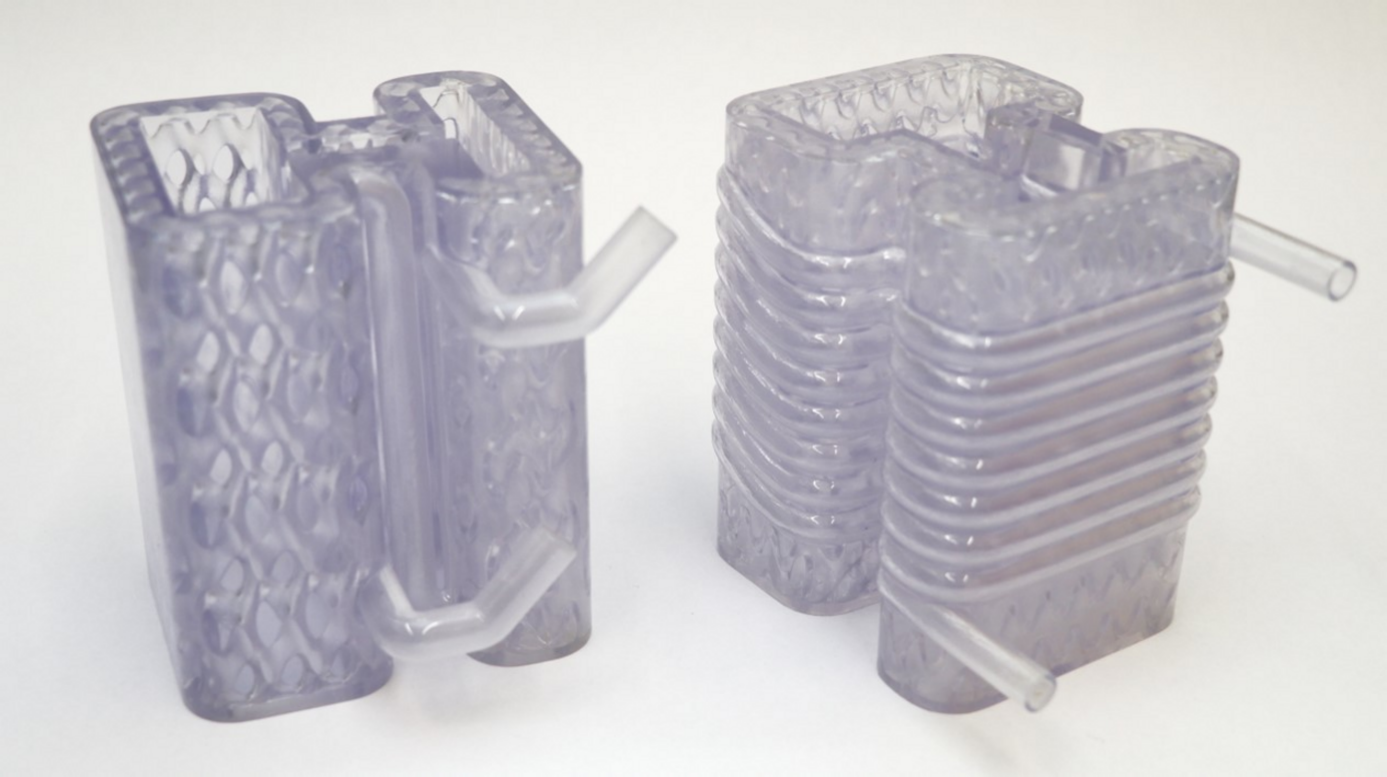
3D printed resin prototypes of the Horizontal and Coil design concepts used for experimental pressure drop measurements.

**Figure 9:**
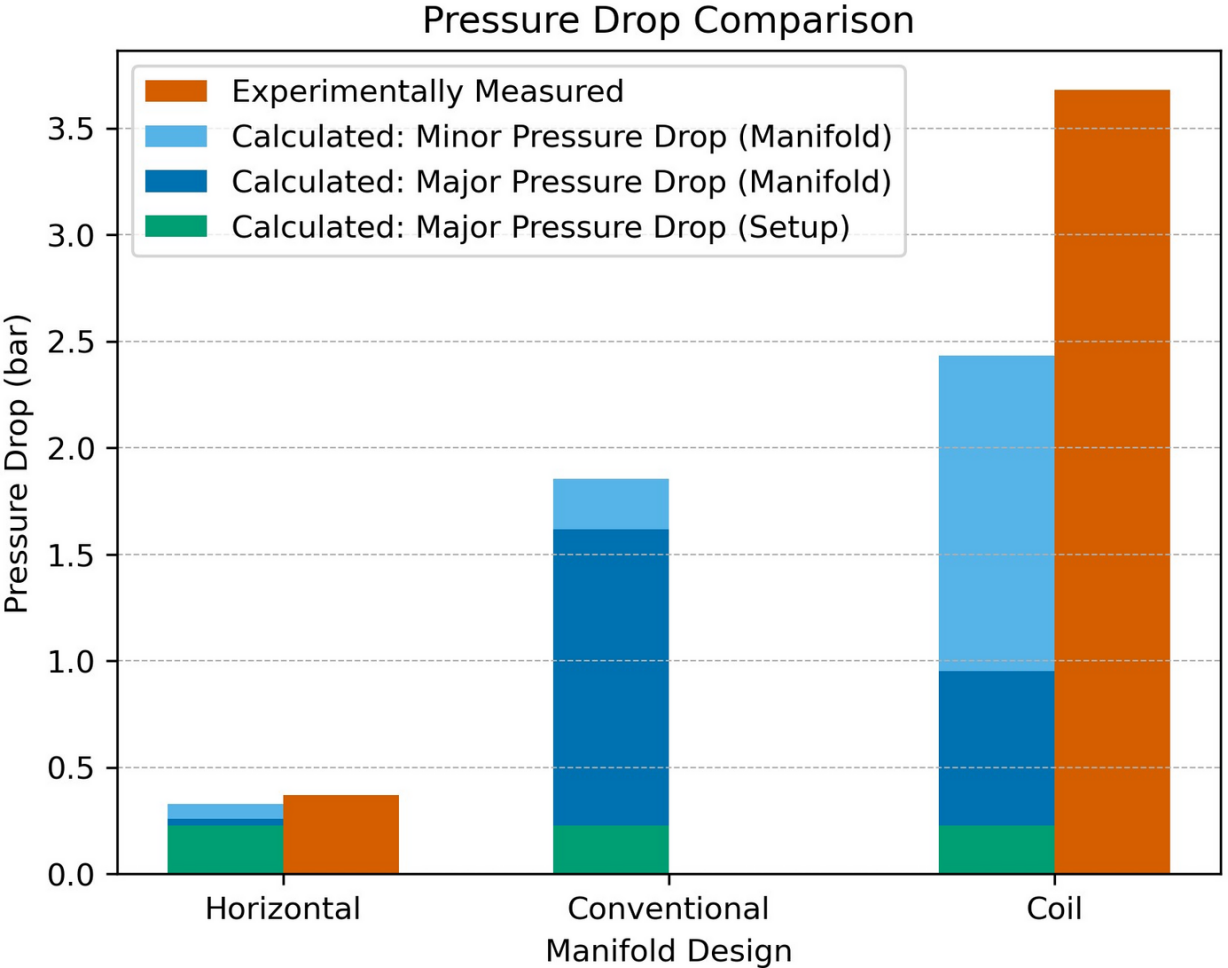
Calculated and experimentally measured pressure drops of the different manifold designs.

The pressure drop was numerically calculated for all three manifolds (conventional, Horizontal, Coil) and experimentally measured for two (Horizontal, Coil) using 3D printed resin prototypes (Fig. 8 ▸). Both the calculation and experiment served as a comparative tool rather than a direct replication of the actual pressure drops anticipated at the synchrotron. The calculated total pressure drop was a combination of major losses and minor losses. Major losses include the manifold and experiment setup pipe lengths, and use the Darcy–Weisbach and Colebrook equations [Appendix *A*[App appa], equations (5) and (6)]. Minor losses cover the cooling pipe geometry bend losses, which were estimated using the equivalent length equation [Appendix *A*[App appa], equation (7)] and minor loss coefficients seen in Appendix *C*[App appc], Table 10. The total calculated pressure drop for the Horizontal, conventional and Coil designs, based on a 6 L min^−1^ flow rate, were 0.33 bar, 1.85 bar and 2.20 bar, respectively, as seen in Fig. 9 ▸ and recorded in Table 12 in Appendix *C*[App appc].

**Figure 10:**
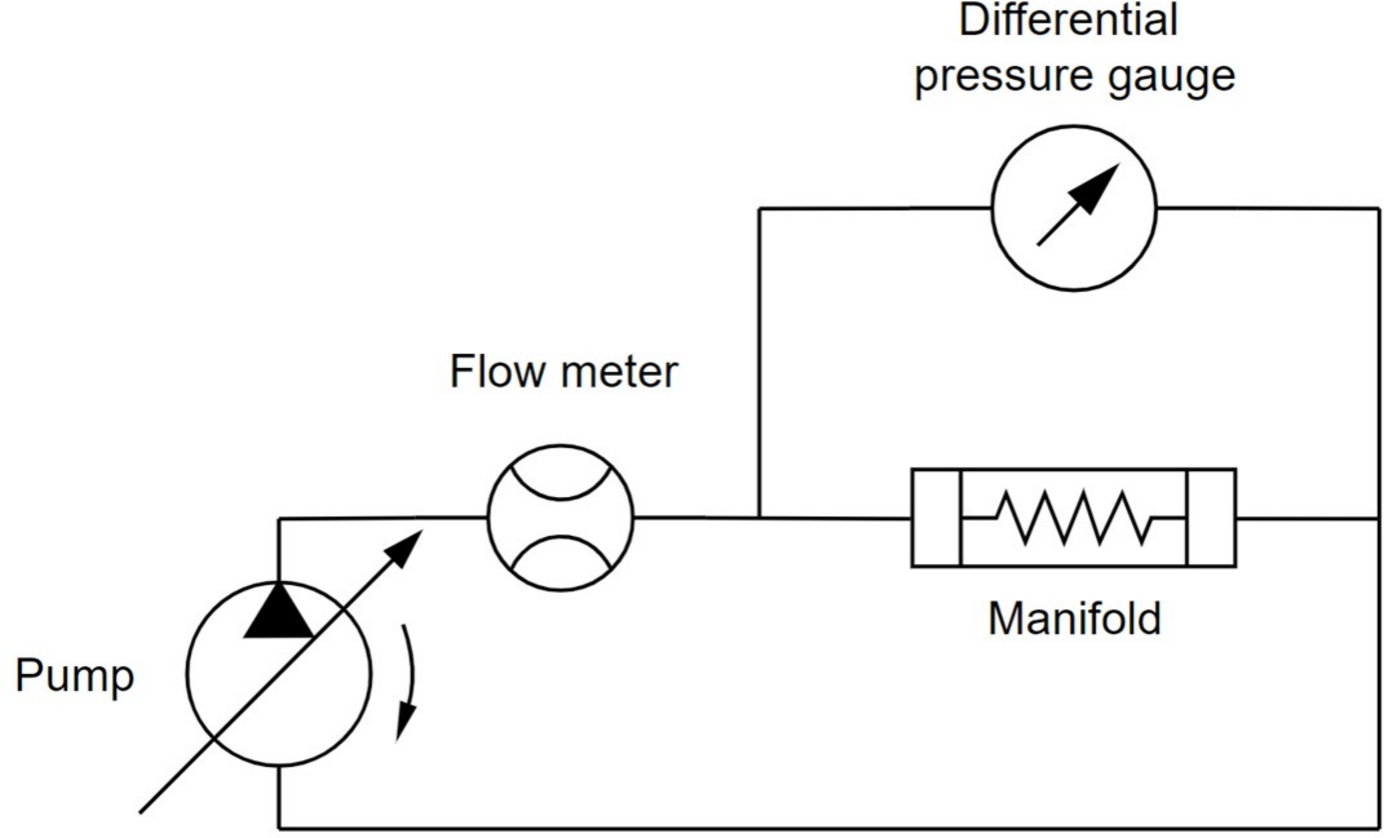
Hydraulic diagram showing the test rig setup used to measure the pressure drop of the Horizontal and Coil designs.

The experimental measurement was conducted using a test rig consisting of a pump (PolyScience DuraChill DCA308D6) with adjustable flow rate and a differential pressure gauge (WIKA 711.12); a hydraulic diagram of the experiment is presented in Fig. 10 ▸. The 3D printed resin models were connected to the test rig, set to a water inlet flow rate of 6 L min^−1^ and fluid temperature of 20°C. The measured differential pressure drop, based on three repeated measurements, was 0.37 bar and 3.68 bar for the Horizontal and Coil designs, respectively, as shown in Fig. 9 ▸. The standard deviation for both measurements was 0.03 bar. The experimentally measured pressure drop for both designs was higher than the numerically calculated value by 12% for the Horizontal and 65% for the Coil design. The higher difference between the calculated and experimentally measured pressure drop in the Coil design can be attributed to unaccounted additional pressure losses, likely caused by the continuous curvature of the helical cooling channels, which induces secondary flows due to centrifugal forces acting on the fluid (Sigalotti *et al.*, 2023 ▸).

The Horizontal design total calculated pressure drop (Appendix *C*[App appc], Table 12) was 82% lower than the intermediate conventional design, while the Coil design calculated pressure drop was 31% higher than the conventional design. The breakdown of the calculated pressure drop into major and minor losses, illustrated in Fig. 9 ▸, provides insight into the primary parameters influencing the pressure pressure drop in each design. For example, the Horizontal and conventional design major losses (major losses of manifold + major losses of set up) accounted for the majority of the pressure drop, as expected. However, in the Coil design, minor losses dominate the measured pressure drop, highlighting how the increasing number of bends was the primary parameter for the higher pressure drop.

To compare the impact of increasing pipe diameter versus reducing pipe length of the Horizontal design, two additional analytical calculations were performed and compared with the conventional design, which has a 5.2 m length pipe and a 6 mm inner diameter (ID). The first calculation maintained the original length (5.2 m) but increased the ID to 8 mm, while the second calculation reduced the length to 0.42 m (similar to the horizontal design) while keeping a 6 mm ID. The results showed a pressure drop reduction of 65% and 69% for the first and second calculations, respectively, compared with the conventional design. This analysis helped isolate the impact of length reduction versus diameter increase, confirming that reducing length in this case, enabled by the design flexibility of AM, had a marginally greater impact on pressure drop reduction than increasing pipe diameter.

### 3.2. AM of benchmark artefacts

**Figure 11:**
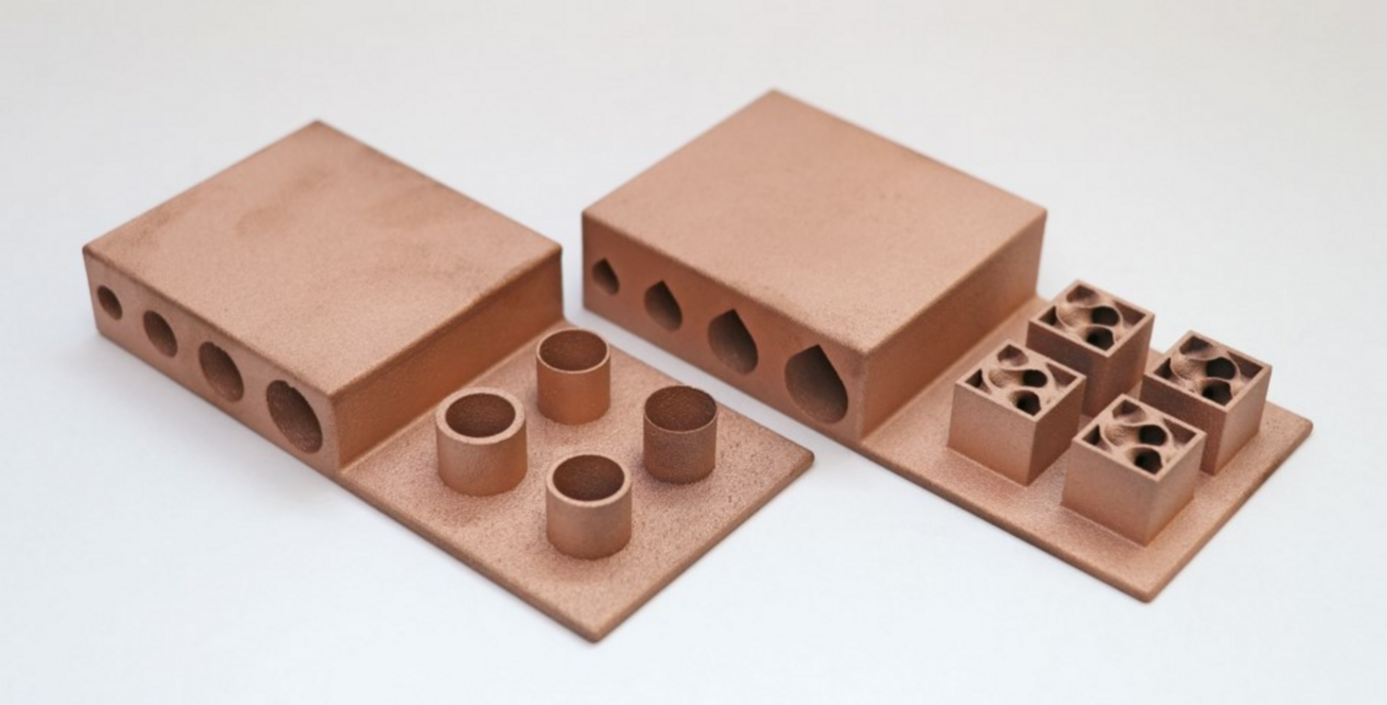
Two custom-made benchmark artefacts with support-less pipes and cylinder wall thickness design (left) and another benchmark artefact with teardrop shape pipes and gyroid wall thickness design (right), both 3D printed in copper, using an EOS M290.

Before 3D printing the Horizontal and Coil absorber design concepts in copper, custom benchmark artefacts were designed and 3D printed in copper to quantify design limitations and to obtain process characteristics relevant to the synchrotron operational environment. Limitations such as minimum wall thickness or need of overhangs can be dependent on multiple factors, for example the geometry, laser print settings or material. To gain an understanding of these design limitations, the first benchmark artefact, shown in Fig. 11 ▸ (left), focuses on investigating the need of AM supports for Horizontal pipes of different dimensions (6 mm-, 8 mm-, 10 mm-, 12 mm-diameter) and their resulting up-skin and down-skin surface roughness. Up-skin or down-skin refer to the surfaces of the printed part that face upward or downward, respectively, during the AM process. The up-skin generally has lower roughness as it is exposed to the open environment during the AM process. In contrast, the down-skin has higher roughness due to partial overlap with unmelted powder, leading to localized overheating and sintered powder adhesion, also known as dross formation (Chahid *et al.*, 2021 ▸). While both up-skin and down-skin can be affected by the staircase effect, roughness in down-facing surfaces is primarily caused by sintered powder buildup. The benchmark artefact also has four cylinders, investigating the print quality of different wall thicknesses (0.25 mm, 0.5 mm, 1 mm, 1.5 mm) representing in this case the absorber cooling pipe wall thickness and part shell thickness [Fig. 18(*c*)].

In the second benchmark artefact, Fig. 11 ▸ (right), the pipes have a teardrop shape, which removes the need for supports. Also, this second artefact includes a gyroid at different wall thicknesses (0.96 mm, 0.64 mm, 0.32 mm, 0.16 mm), representing the absorber infill thickness, seen in Fig. 18(*c*).

The benchmark artefacts were 3D printed using an EOS M290 industrial-grade LPBF AM system using recycled high-purity (>99.6%) copper powder. The EOS M290 machine features a standard 400 W infrared laser system with a 100 µm spot size where the LPBF process is carried out in an argon atmosphere at oxygen content below 0.1% and a build plate temperature of 35°C. Following printing, all benchmarks were annealed following the powder supplier recommended heat treatment cycle of 1000°C for one hour in an inert atmosphere. The annealing step leads to larger microstructure grain sizes expected to enhance thermal conductivity, as reported in a previous copper-based alloy study (Biffi *et al.*, 2024 ▸). The benchmarks were then removed from the build platform using EDM and surface finished using glass bead blasting. LPBF print parameters used for manufacture are listed in Table 2 ▸.

### 3.3. Surface roughness metrology

**Figure 12:**
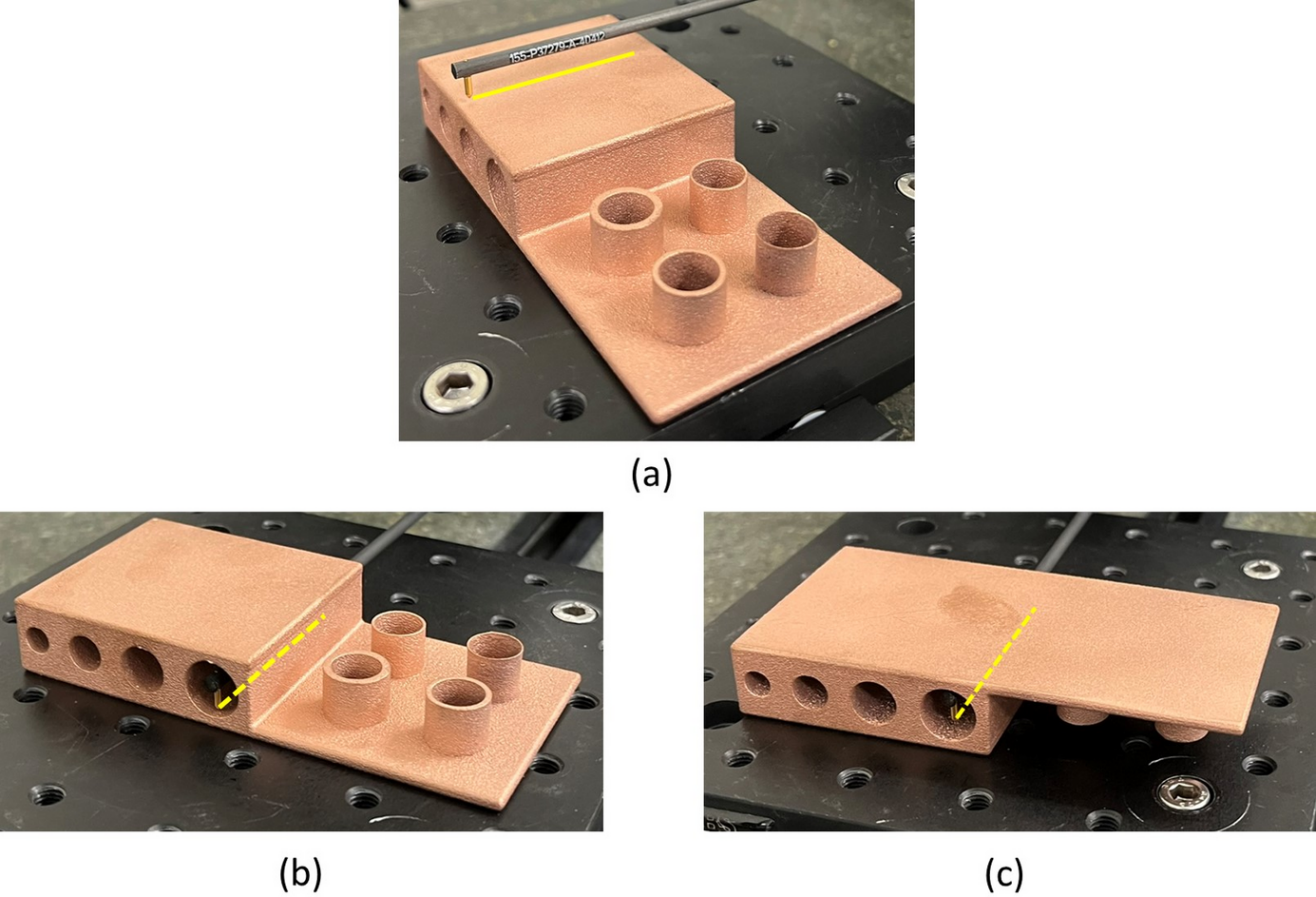
Profile surface roughness measurement of the outer upper surface (*a*), up-skin of the 12 mm pipe (*b*), and down-skin of the 12 mm pipe (*c*).

Surface roughness of the first benchmark artefact, Fig. 11 ▸ (left), was measured using a contact profilometer (Taylor Hobson Form Talysurf) using a 2 µm diamond stylus tip. The assessed lengths included the benchmark artefact upper outer surface, and the down-skin and up-skin of the 12 mm diameter hole, as seen in Fig. 12 ▸. The average surface roughness (*Ra*) and mean peak to valley height (*Rz*) were obtained from three repeated measurements, using an evaluation length of 40 mm and a cut-off length of 8 mm, in accordance with ISO 4288 (ISO, 1996 ▸). Due to the large size of the stylus shank clearance (distance from stylus tip to stylus arm) with respect to the circular pipes, only the largest hole diameter of 12 mm was measured. The measurement results indicated an *Ra* and standard deviation (in parentheses) of 7.47 µm (0.04 µm) on the outer surface, 19.26 µm (0.59 µm) on the down-skin and 16.77 µm (0.71 µm) on the up-skin. The measurement results also indicated an *Rz* and standard deviation (in parentheses) of 38.39 µm (0.53 µm) on the outer surface, 91.28 µm (1.6 µm) on the down-skin and 85.84 µm (2.51 µm) on the up-skin.

### 3.4. Dimensional metrology

**Figure 13:**
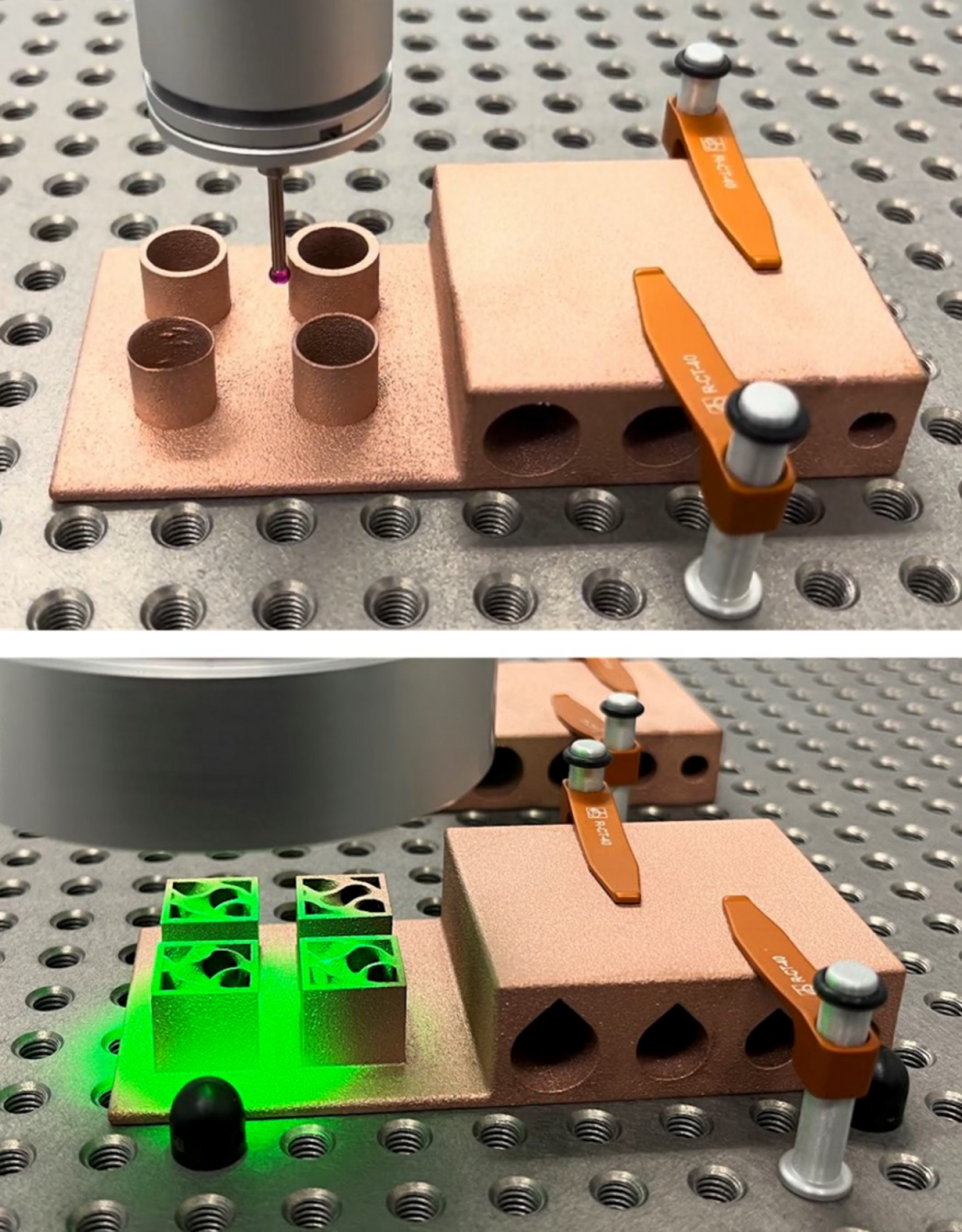
Cylinder wall thickness measurement using CMM stylus (top), and gyroid wall thickness measurement using CMM camera (bottom).

**Figure 14:**
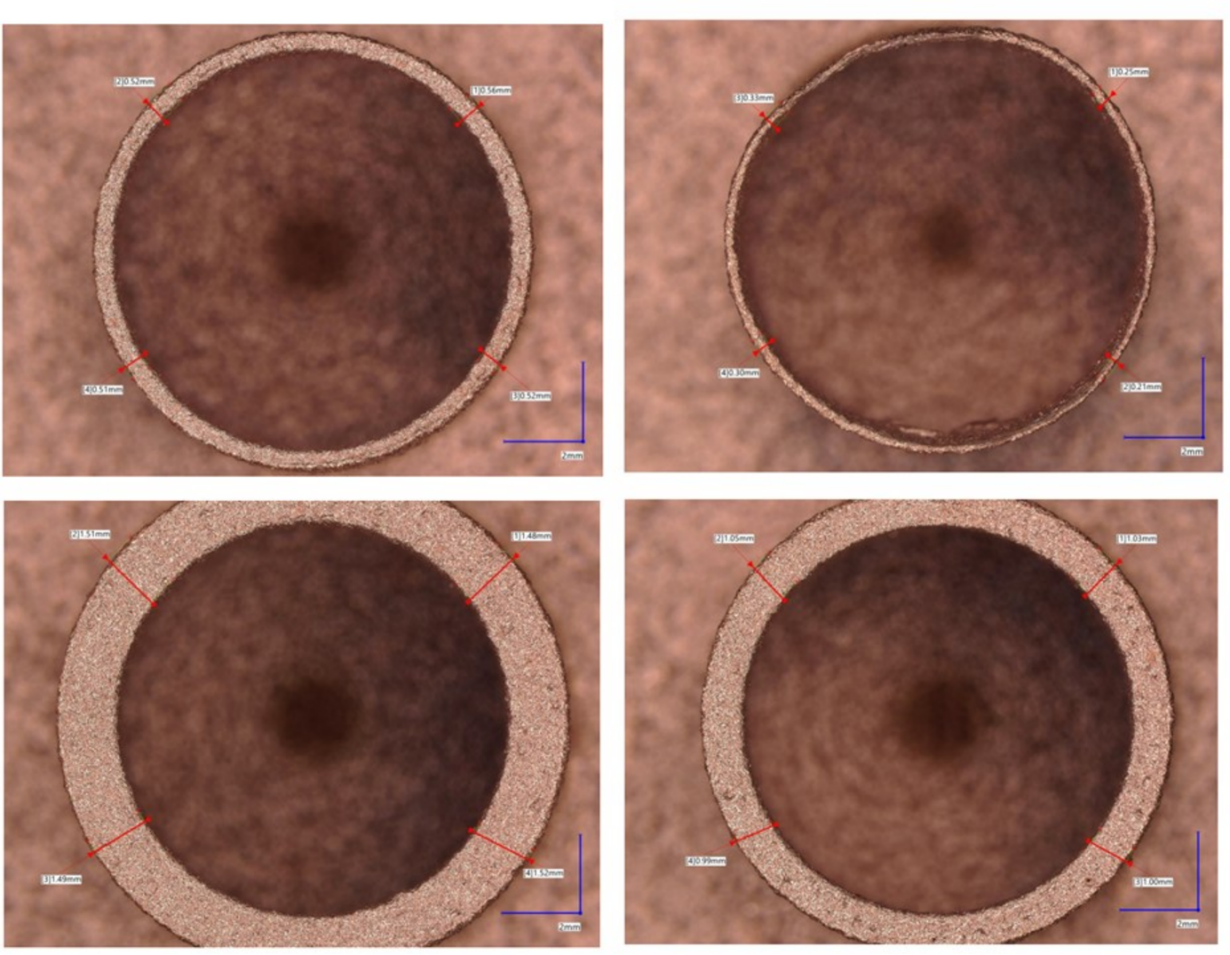
Microscope images showing the different contour forms of the cylinders with wall thicknesses of 0.25 mm, 0.5 mm, 1 mm and 1.5 mm (from top right to bottom left). These cylinders served as a reference to guide the selection of the pipe wall thickness and shell wall thickness for the copper printed absorber.

**Figure 15:**
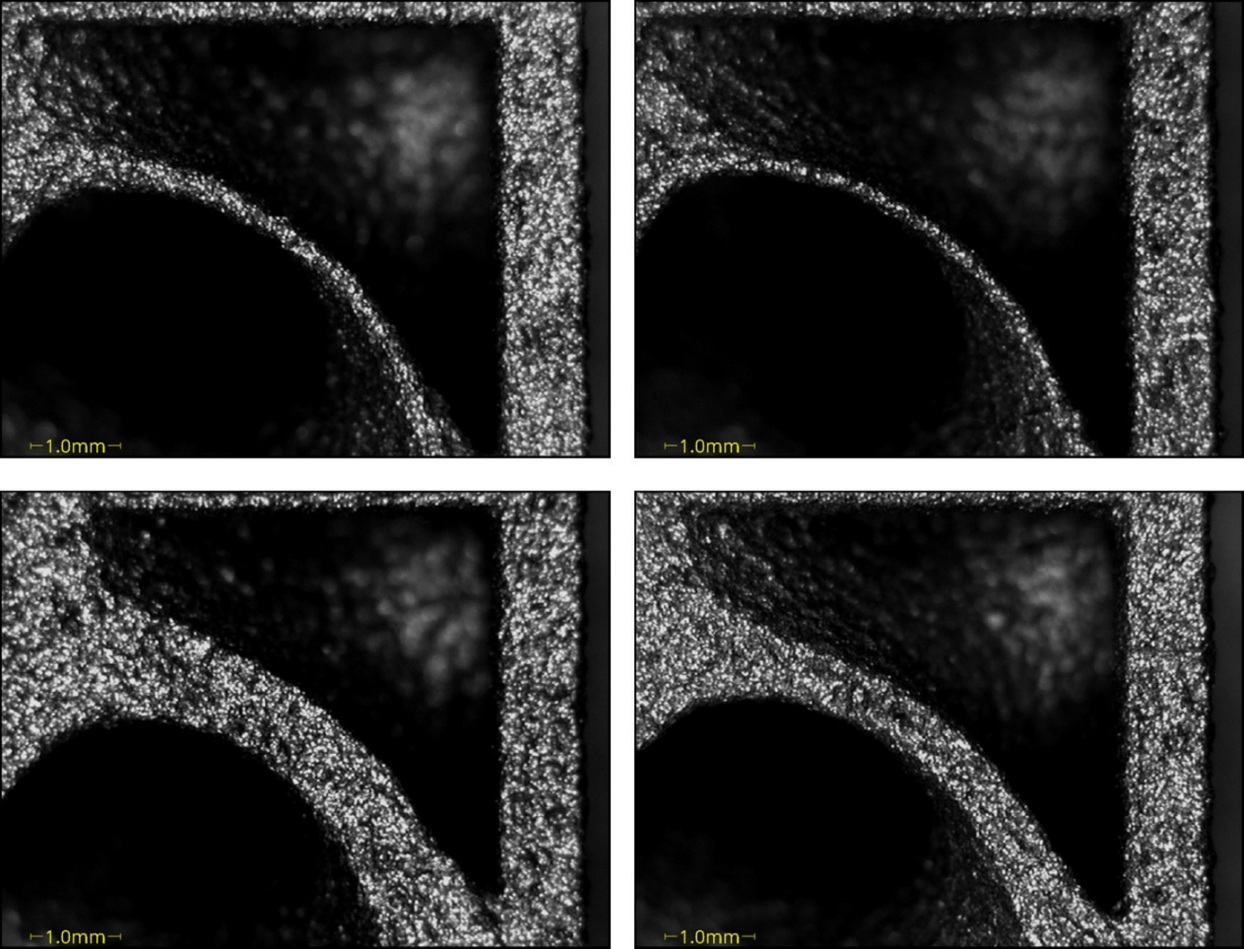
CMM camera images showing gyroids with wall thicknesses of 0.16 mm, 0.32 mm, 0.64 mm and 0.96 mm (from top right to bottom left). These gyroids served as a reference to guide the selection of the infill wall thickness for the copper printed absorber.

Dimensional measurements were taken using a coordinate measuring machine (CMM; Hexagon 7:10:7). On the first benchmark artefact (Fig. 11 ▸, left); each extruded cylinder wall thickness was measured using 15 outer-diameter (OD) points and 15 ID points, repeated three times for a sample standard deviation. The cylinder with the smaller wall thickness of 0.25 mm was challenging to measure due to an uneven contour; therefore, only 8 points were measured instead of 15 points. The CMM used a 3 mm-radius ruby ball tip; the equipment set-up can be seen in Fig. 13 ▸ (top). A close-up image of the cylindrical features can be seen in Fig. 14 ▸. On the second benchmark artefact (Fig. 11 ▸, right) the gyroid infill wall thickness was determined using the CMM HP-C vision sensor camera, and the setup is shown in Fig. 13 ▸ (bottom). From each CMM captured gyroid image (Fig. 15 ▸), the user performed three repeated wall thickness measurements perpendicular to the surface of each gyroid using the ruler tool in *Fiji* software, after scaling based on the CMM scale bar (Schindelin *et al.*, 2012 ▸).

The CMM measured cylinder wall thickness for the 1.5 mm, 1 mm, 0.5 mm and 0.25 mm was 1.56 mm, 1.07 mm, 0.56 mm and 0.32 mm, respectively, with a standard deviation of zero for all measurements at two decimal places. Also, the CMM measured gyroid wall thickness and standard deviation for the 0.96 mm, 0.64 mm, 0.32 mm and 0.16 mm was 0.86 mm (0.02 mm), 0.52 mm (0.01 mm), 0.29 mm (0.03 mm) and 0.21 mm (0.02 mm), respectively. These measurements show a maximum dimensional deviation from CAD of 0.07 mm for the wall thickness of a cylinder and 0.12 mm for the wall thickness of a gyroid. Both wall thickness measurements can be seen in Tables 3 ▸ and 4 ▸.

### 3.5. Porosity analysis

**Figure 16:**
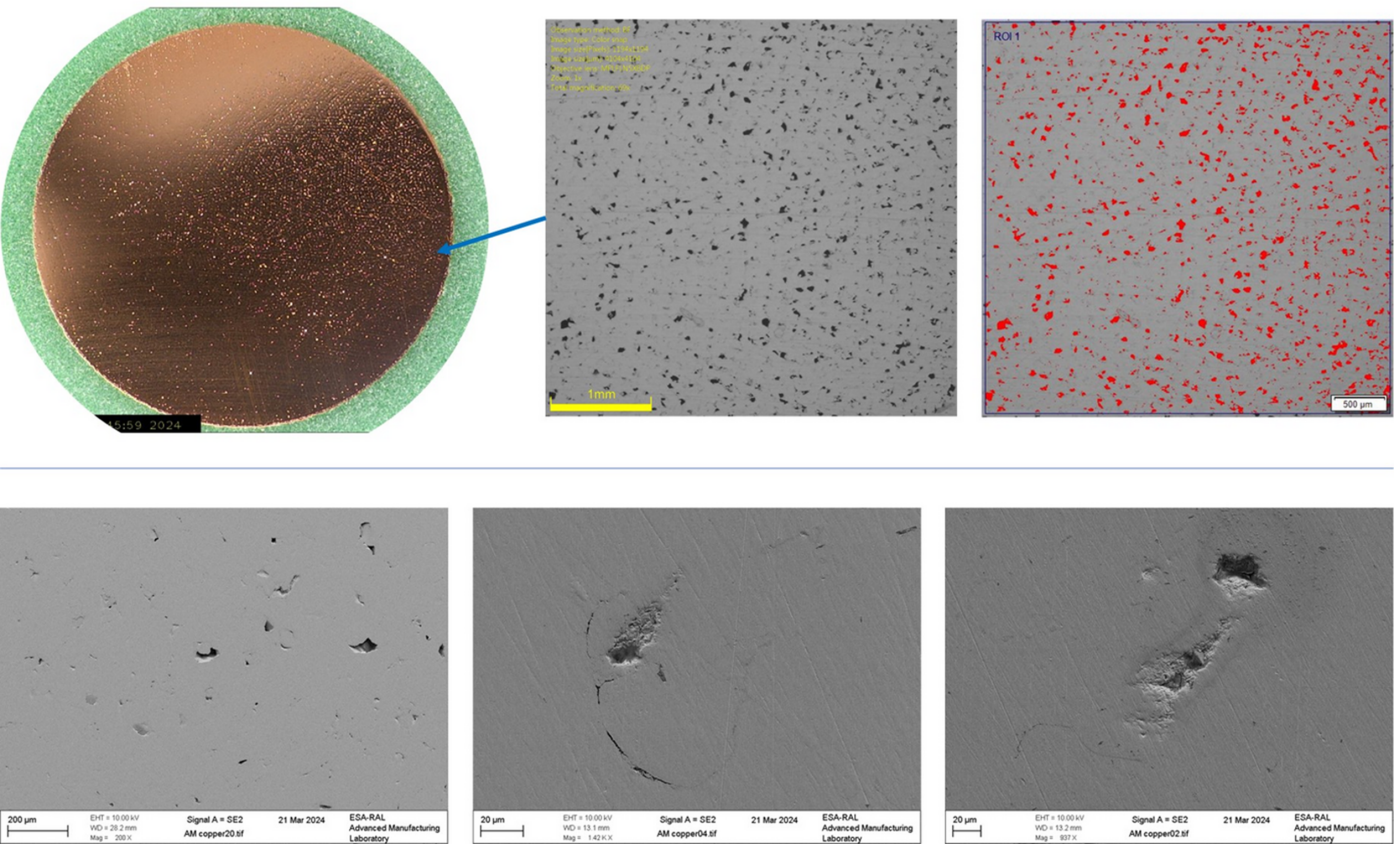
Porosity analysis performed on a ground and polished 18 mm-diameter sample, revealing a 6.20% porosity using a microscope (top), and the shape of different pores using an SEM (bottom).

A solid copper cylinder, 18 mm in both diameter and height, was 3D printed for density evaluation with the cylinder’s *z*-axis (height) printed perpendicularly to the build plate. The cylinder was then halved in the horizontal plane parallel to the build plate and one of the cross sections was ground using successive steps from 320 to 4000 grit and then polished with a slurry containing 3 µm abrasive particles using oxide polishing suspension (OPS) to achieve a smooth finish that revealed the surface pores. The polished surface was measured using an optical microscope at ×65 magnification (DSX510 Olympus). As seen in Fig. 16 ▸ (top right), an ROI of 4 mm × 4 mm was captured and binarized using an automatic threshold tool in *Olympus Stream* image analysis software, revealing a porosity percentage of 6.20%. A scanning electron microscope (SEM) (Zeiss FESEM LEO1530) was used to evaluate the morphology of the pores, using three magnifications ranging from ×200 to ×1420, seen in Fig. 16 ▸ (bottom). X-ray computed tomography (XCT) was applied on the cylindrical porosity sample but different factors such as the high density of copper, beam hardening artefacts and relatively small size of pores meant that the XCT scan slices did not show porosity. The applied cross section microscope measurement, although destructive and only taking measurements from a single plane, can deliver relatively more reliable results compared with XCT (Chahid *et al.*, 2024 ▸).

The pores on the sample surface were unevenly distributed (Fig. 16 ▸, top left), suggesting a recoater anomaly. Recoaters in LPBF AM machines are responsible for depositing and evenly spreading the feedstock powder, ensuring a compact layer. This anomaly, assuming a right-to-left build direction, may have caused a powder flow issue when the recoater first contacted the previously exposed layer. The SEM images (Fig. 16 ▸, bottom) reveal irregular-shaped porosity, indicative of lack of fusion (Kotadia *et al.*, 2021 ▸). This type of porosity usually results from insufficient energy in the powder bed, typically addressed by increasing laser energy, especially with copper. However, the localized porosity in this case, found alongside dense material processed with the same laser parameters, indicates inadequate powder delivery rather than insufficient laser power.

### 3.6. AM of prototypes in copper

**Figure 17:**
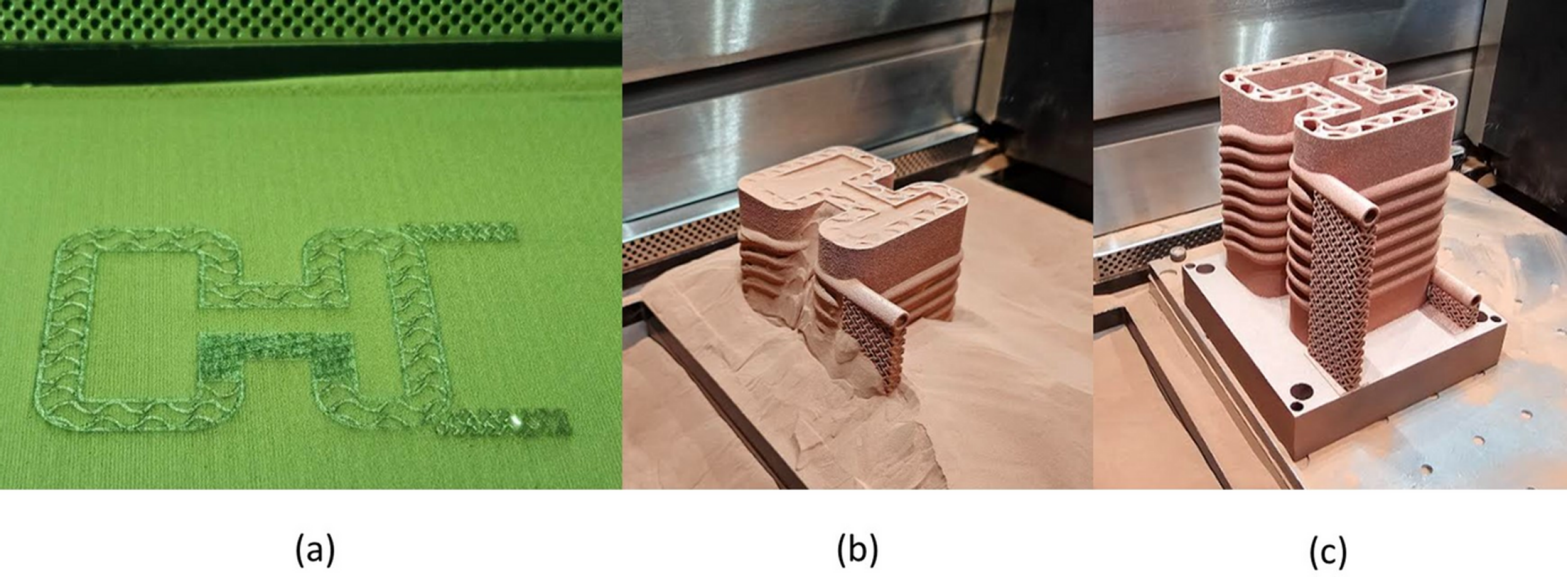
LPBF process (*a*), lightweight Coil pipe concept design within the powder bed (*b*) and without surrounding powder (*c*).

**Figure 18:**
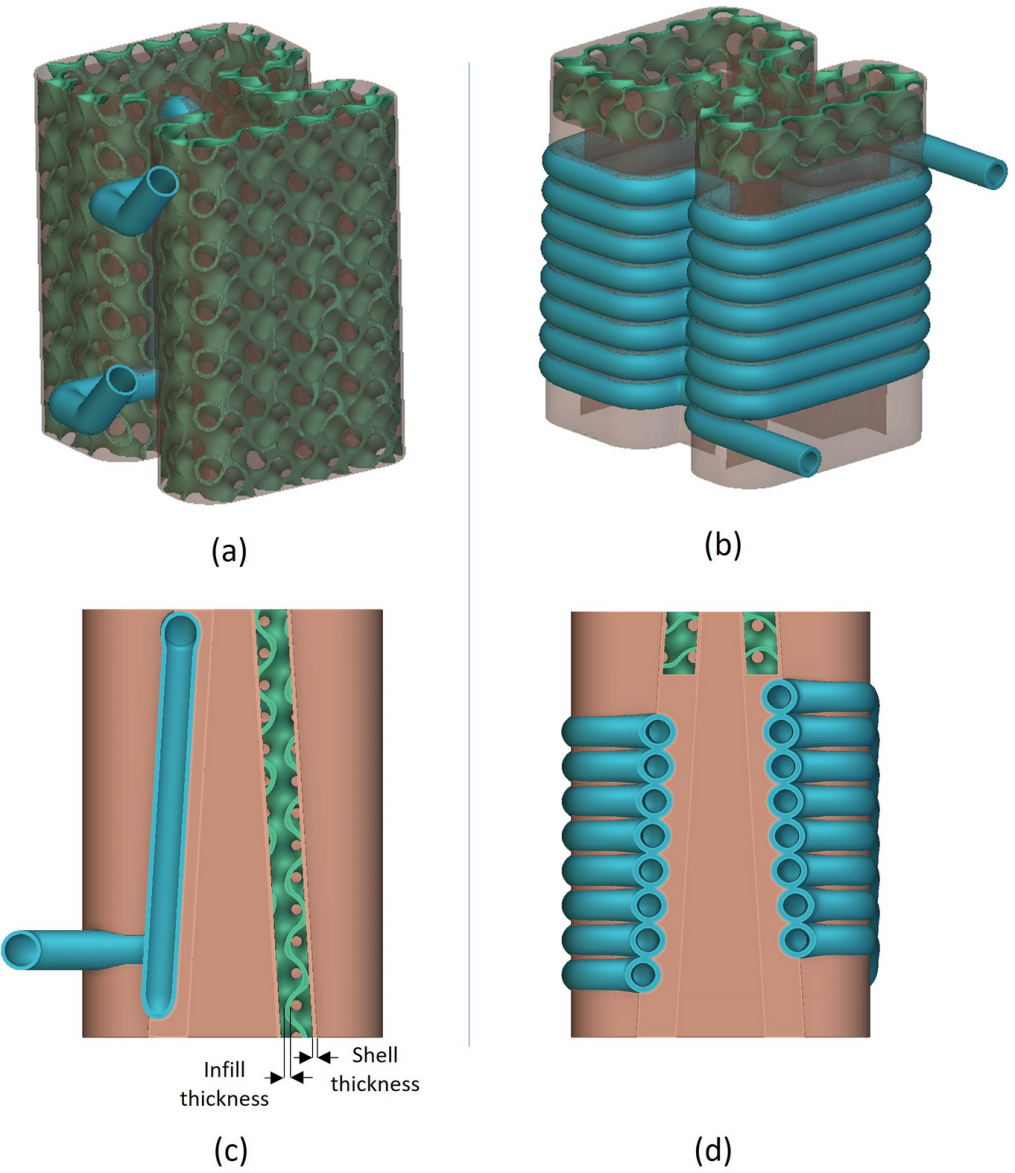
Transparent CAD model of lightweight Horizontal design (*a*) and lightweight Coil design (*b*) showing the gyroid infill in green and cooling channel in blue. Cross section of the lightweight Horizontal (*c*) and lightweight Coil design (*d*) showing how the gyroid infill covers the majority of the lightweight Horizontal design but only the upper 16 mm of the lightweight Coil design.

**Figure 19:**
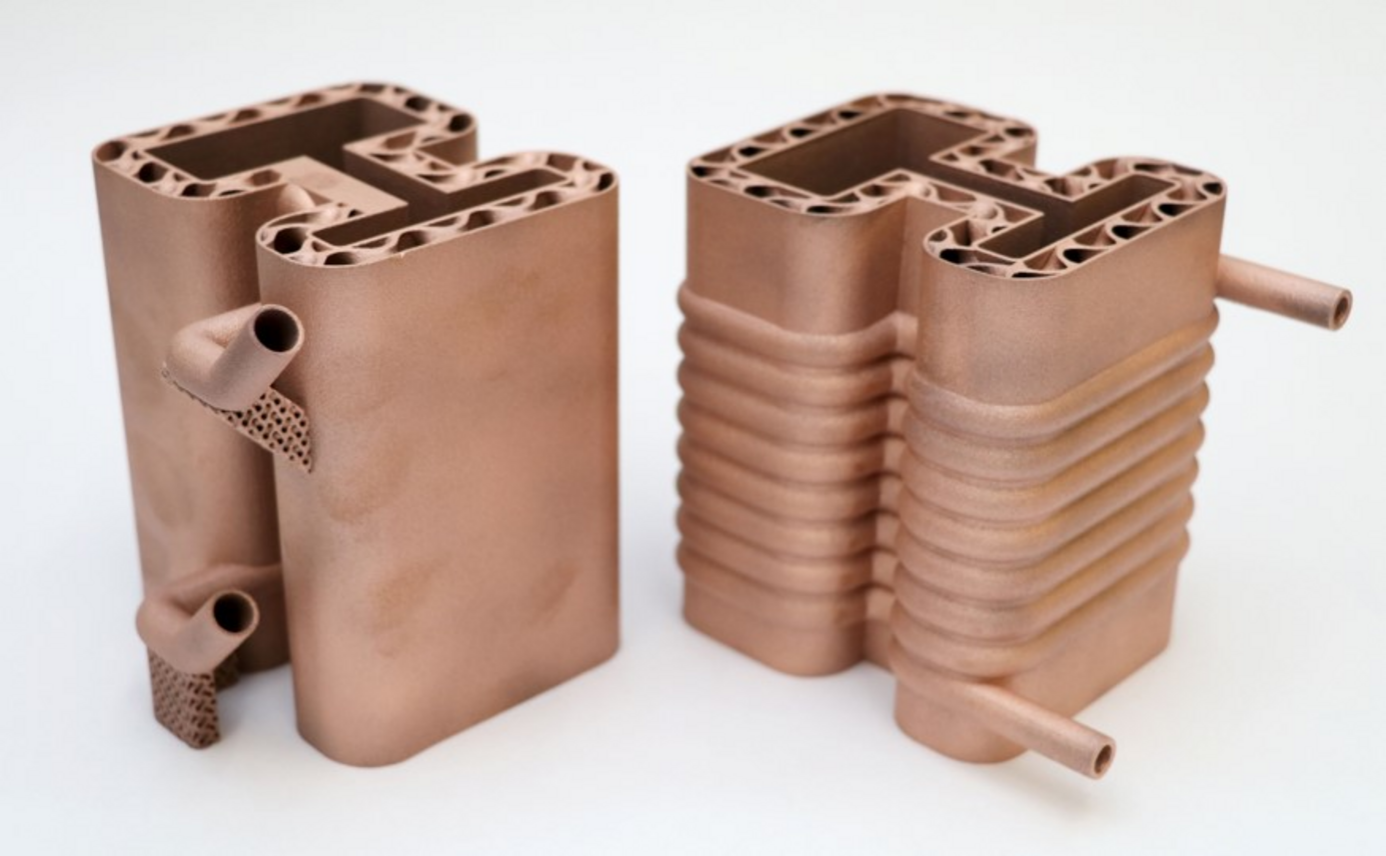
Additive manufactured lightweight Horizontal absorber (left), and lightweight Coil (right) shown in the same orientation as the upward print direction.

The benchmark artefacts provided insights into the print quality of different geometries, informing further development of the two concept designs (Horizontal and Coil) prior to copper printing. For example, it was evident from Fig. 11 ▸ (left) that the 6 mm-diameter and 8 mm-diameter pipes do not require supports or redesigning into a teardrop shape. Furthermore, the benchmark artefact cylindrical and gyroid features led to selecting a 1 mm gyroid infill wall thickness and a 1 mm shell wall thickness within each absorber design. The Horizontal design had a 1 mm pipe wall thickness while the Coil design had a 1.5 mm pipe wall thickness chosen for additional strength during the printing process of the inlet and outlet region seen in Fig. 17 ▸. Finally, non-functional features of the intermediate absorber design [Fig. 3 ▸(*c*)], in this case the external cylindrical geometry inherent of the conventional manufacturing, were removed and replaced by fully dense regions in contact with the BTS and gyroid infill regions in the remainder of the design, as seen in Figs. 18 ▸(*c*) and 18(*d*) and named in the remaining of this study as lightweight Horizontal and lightweight Coil. The removal of the absorber cylindrical geometry and using shells plus infills led to a material waste and mass reduction of the two concept designs. The mass of the lightweight Horizontal and lightweight Coil 3D printed models (Fig. 19 ▸), measured by a weighing scale, were 1.4 kg and 2.3 kg, respectively, while the mass of the intermediate conventional design, seen in Fig. 4 ▸ (bottom), is estimated to be 10.1 kg. The mass of the intermediate conventional design is calculated using the CAD volume data and copper density. This means that in comparison with the intermediate conventional model (Fig. 4 ▸, bottom) a mass saving of 86% and 77% was achieved by using a lightweight Horizontal and lightweight Coil design, respectively. The mass difference between the Horizontal and Coil design is attributed to the limited upper use of gyroid infill in the lightweight Coil model (Fig. 18 ▸) compared with a gyroid infill that runs all the way through in the lightweight Horizontal model (Fig. 18 ▸).

**Figure 20:**
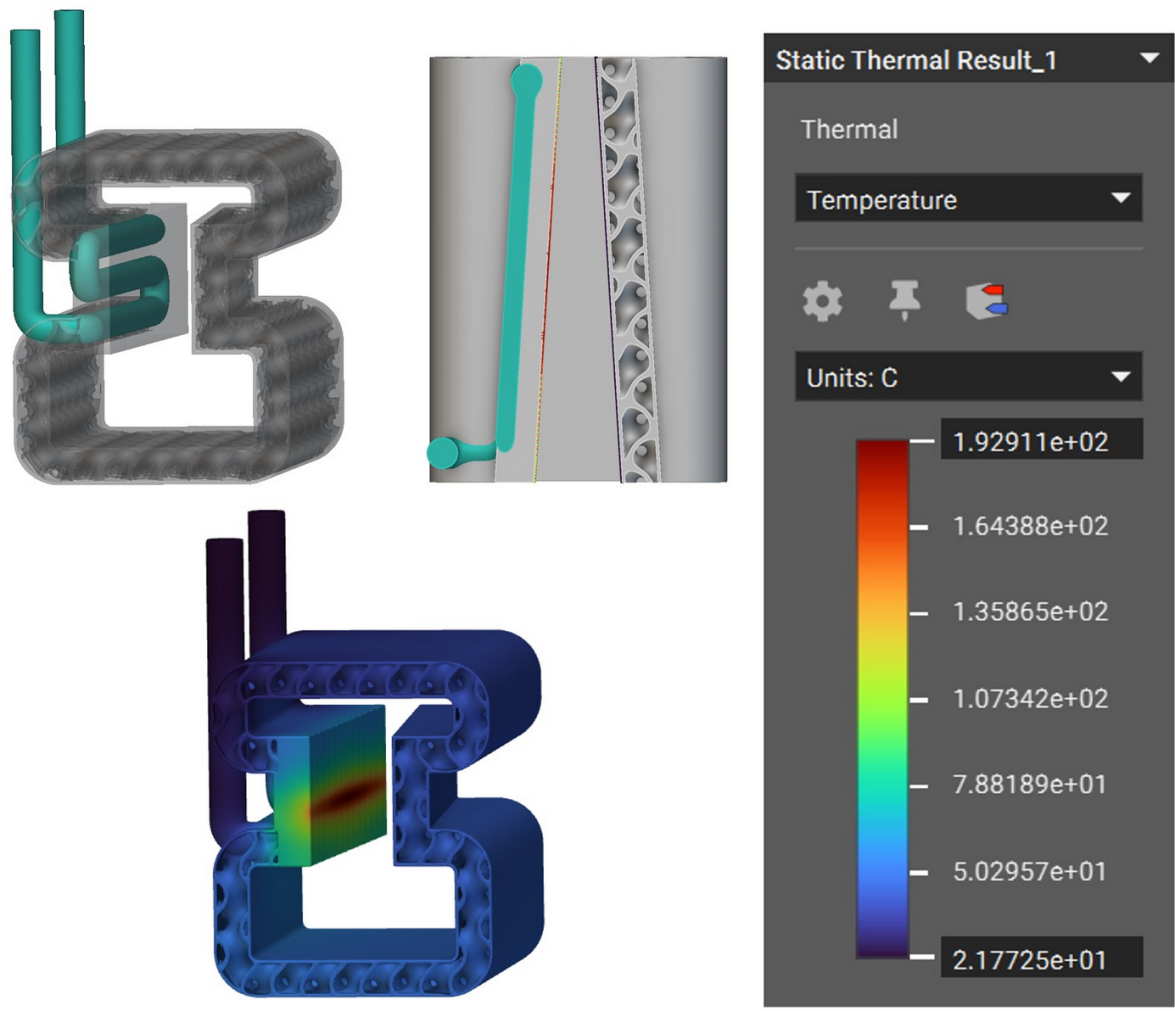
Lightweight Horizontal model thermal simulation and cross section.

To assess the impact of lightweighting on heat transfer, linear steady state thermal simulation was also applied on the lightweight Horizontal AM design, favoured in this study for having the lowest pressure drop. Horizontal pipe geometry and boundary conditions are identical to the ones used for Fig. 6 ▸ (top) and shown in Table 1 ▸ allowing for a direct assessment of the impact of lightweighting on heat transfer. The resulting maximum temperature was 192.9°C as seen in Fig. 20 ▸. This result shows an 11% lower maximum temperature than the intermediate conventional model simulation (Fig. 4 ▸, bottom), as summarized in Table 5 ▸. This result shows how lightweighting and use of gyroids has increased the maximum temperature by 4.2%. This can be attributed to the reduced material available for heat conduction between the BTS and cooling channel surfaces. This can be mitigated by further increase of the solid volume of the BTS seen in Fig. 18 ▸, until the desired balance is achieved between mass reduction and heat transfer. While mass reduction is not a primary aim in this study, lightweighting in AM can significantly reduce raw material usage and print time, impacting cost and eventually AM adoption.

Steady state thermal simulations assume a constant water temperature and do not account for dynamic variations that may influence the thermal performance of the absorber. While this study uses steady state thermal evaluation of an AM absorber as a comparative tool with a conventional counterpart, future investigations will incorporate transient thermal simulations to better capture dynamic temperature variations and assess deviations between simulation and experimental results, further refining the accuracy and applicability of the proposed AM absorber design and future ones.

To assess porosity, a gross leak test was conducted on the two copper prototypes by sealing both the inlet and outlet of the manifold with insulating rubber end caps and submerging each prototype underwater. Air bubbles were observed escaping from the surface of the pipe manifold, indicating leaks and compromising the sealing integrity of the prototypes. This confirms that the porosity level of 6.20% must be reduced in future prototypes, following recommendations given in Section 4.

Part consolidation was achieved with the two AM designs. Compared with the intermediate conventional model, the part count was reduced from 21 individually brazed pipes into a single manifold. This reduction decreases the time and cost associated with joining each individual pipe to the absorber design, while potentially reducing points of failure caused by vacuum brazing.

During the AM process, both model designs required supports for the pipe inlet and outlet. These supports were retained for the lightweight Horizontal model but removed for the lightweight Coil model. The LPBF process print settings were identical to those listed in Table 2 ▸, and an image of the process steps is shown in Fig. 17 ▸. The resulting prototypes are displayed in Fig. 19 ▸.

## 4. Discussion

This study introduced and evaluated two AM conformal concept designs of an intermediate absorber model. Notable improvements were observed through thermal simulation, as well as experimental and numerical calculations of pressure drop.

In terms of the primary system level aim of the study focusing on increasing heat transfer, the thermal simulation results showed that the Coil design achieved the most significant temperature drop of 20.0%. This result can be attributed to a reduction in the distance between the cooling channels and the BTS, which is enabled by the use of AM and its associated increased design freedom. The shorter distance increases the conduction temperature gradient d*T*/d*x* shown in Appendix *A*[App appa], equation (8), leading to a higher temperature difference between a hotter pipe wall and coolant, providing a higher rate of heat transfer. Another factor is the densely placed conformal pipe design, which led to an increase of the convection surface area *A*_s_ shown in Appendix *A*[App appa], equation (9), also leading to a higher heat transfer. An improved temperature drop was also obtained using the Horizontal design (15%), also attributed to reducing the distance of the cooling channels to the BTS. This reduction in the distance, from ∼20 mm in the conventional to 5.7 mm for the Coil design and 4.7 mm for the Horizontal design, could not have been achieved by conventional manufacture. Conventionally drilling cooling channels closer to the BTS would expose them to the internal vacuum pockets positioned above and below the BTS (Fig. 4 ▸, bottom). Furthermore, the lightweight Horizontal design temperature drop was limited to 11%, highlighting the impact of reducing material available for heat conduction between the BTS and cooling channel surfaces. In the thermal simulation, the AM material was assumed to have identical thermal conductivity as the conventionally manufactured OFHC copper. Future study will need to experimentally measure the thermal conductivity of the produced AM components and compare its performance with OFHC copper.

Also part of the system level aim in this study was the pressure drop, of which the lightweight Horizontal design achieved the lowest value, calculated to be 82% lower than that of the intermediate conventional design. This is largely attributed to the reduction in pipe length, shortened from 5.2 m of the conventional design to 0.42 m for the Horizontal design (Appendix *C*[App appc], Table 8). Pipe length *L* is a multiplier in the pressure drop equation [Appendix *A*[App appa], equation (5)] and had a higher impact on the conventional design major losses compared with minor losses caused by pipe bends (Fig. 9 ▸).

Although the Horizontal and lightweight Horizontal design cool the absorber from only one side and result in a lower temperature drop than the Coil design, they are still favoured between the two concepts. This is due to the combination of the positive thermal simulation and pressure drop results suggesting that the lightweight Horizontal design has 11% lower maximum temperature and 82% lower pressure drop than the conventional design. In this case, absorbers that require cooling of both sides of the BTS will eventually need a horizontal pipe in each side of the absorber.

When applying the findings of this primary aim on the full size absorber, improved heat transfer and reduction of maximum temperature improves the thermal fatigue life and allows for shorter absorbers that are more efficient. Also, a reduced pressure drop decreases the pumping requirements, leading to energy savings and improved system stability. This reduction in pumping load decreases vibrations, both at the pump level, helping to prevent premature mechanical fatigue failure, and also at the absorber level, where excessive vibrations could affect the performance of nearby sensitive equipment. This is important for the next generation of synchrotrons, which require nanometre-level beam stability (Simos, 2019 ▸; Cacho-Nerin *et al.*, 2020 ▸).

In terms of the secondary aim, investigating the compliance of AM with regard to the absorber operational requirements, the benchmark artefacts were relevant in understanding the process limitations, simplifying the measurement process and planning of the absorber prototype design features, thereby minimizing time and cost associated with print failures.

For example, the measured surface roughness, although glass bead blasted, did not meet the specified finish requirement of *Ra* ≤ 1.6 µm for vacuum-facing surfaces. High surface roughness can cause increased photon scattering leading to localized heating of surfaces in the surrounding areas and possibly lead to higher degassing times. To achieve a lower surface roughness, future study can investigate the use of other mechanical and chemically assisted finishing processes (Torims *et al.*, 2023 ▸) for the BTS, but also for the internal cooling channels to ensure the removal of semi-sintered AM powder. The reported surface roughness of the copper circular cooling channels is expected to cause a higher pressure drop compared with the teardrop-shaped channels, as documented in the literature (Wildgoose *et al.*, 2024 ▸). This is because teardrop-shaped cooling channels have been found to exhibit lower surface roughness and friction factors, leading to a reduced pressure drop.

Furthermore, dimensional metrology results showed a maximum deviation from CAD of 0.07 mm and 0.12 mm for cylindrical or gyroid feature wall thickness, respectively. This deviation was satisfactory for this study; however, a full size absorber will include critical features like BTS alignment or flange interfaces, likely requiring EDM and computer numerical control (CNC) machining for improved dimensional accuracy. While ID dimensional measurements of the horizontally printed cooling channels were not performed, significant dimensional deviations and uncertainties are expected due to the relatively large peak-to-valley variations, as indicated by the *Rz* values reported in Section 3.3. In addition, porosity analysis revealed that the majority of the pores were caused by a lack of fusion. The scan path incorporated rotation, removing directionality and making it unlikely that the pore distribution is influenced by the scanning pattern. A gross leak test on the two AM prototypes, which are assumed to incorporate the measured porosity value of 6.20%, confirmed the presence of leaks, compromising their sealing integrity and emphasizing the need for porosity reduction methods. While the main reason for porosity was attributed to powder delivery rather than laser settings, which were seen to deliver denser regions in the cross section of the part, future study can investigate the use of *in situ* meltpool measurements to ensure track overlap and a hard recoating blade instead of the used brush blade, which can deliver parts with less porosity (Robinson *et al.*, 2022 ▸). An alternative is to investigate the use of hot isostatic pressing (HIP) to reduce porosity of AM copper parts. This is important because a high level of porosity can negatively impact thermal conductivity and potentially lead to inefficient degassing of the absorber, which operates at an ultra high vacuum (UHV) typically less than 10^−9^ mbar. Although not sufficient to prevent leaks, the measured porosity of 6.20% is an improvement over the ∼12% reported in the literature using low-power infrared laser processing (Robinson *et al.*, 2022 ▸). In contrast, high-power infrared lasers exceeding 600 W have been shown to achieve porosity levels as low as ∼3% (Colopi *et al.*, 2018 ▸).

Finally, although the dimensional, surface roughness and porosity analysis results highlighted how post-processing steps are currently inevitable and require further research, investigating AM highlighted benefits such as lightweighting, consolidation and a potential reduction into manufacturing lead time. For example, the applied lightweighting led to an 86% mass reduction between the lightweight Horizontal and intermediate conventional design and a part consolidation from 21 individually brazed pipes into a single manifold. Removing the vacuum brazing operation of each individual pipe bend can lead to higher heat transfer (by removing thermal resistance from brazed joints), reduced points of failure and reduced complexity and part count. For example, the lead time of the AM and post-processing of each intermediate absorber design concept took ∼1.5 weeks to complete (printing, annealing, EDM and bead blasting). By estimating four weeks for the AM and post-processing of an optimized full-size absorber, and further machining and surface treatment, estimated to take another four weeks, the estimated AM time for a full size absorber becomes about two months, which is less than half the current about five months manufacturing lead time for conventional DLS absorbers.

## 5. Conclusion

In this paper, the primary system level aim was to investigate the effectiveness of conformal cooling geometries and their impact on heat transfer and fluid pressure drop, two factors impacting the size, performance and efficiency of high heat load photon absorbers. The secondary aim was to investigate the compliance of low power infrared laser AM process in producing copper absorbers for a synchrotron environment.

Findings of the primary aim revealed that conformal cooling channels can lead to maximum temperature drops of 11%, suggesting the potential to increase the BTS angle, allowing for shorter absorber components. Facility scale synchrotrons like DLS incorporate ∼100 absorbers, and reducing their size would provide valuable additional space. This space could be better utilized for more critical components that enhance the efficiency, precision and accuracy of the facility, meeting the increasing demands for improved beam performance. The primary aim findings indicated that, while some conformal designs have improved heat transfer, they can be disadvantaged by increased pressure drop. This was overcome by choosing a conformal geometry that minimizes pipe length, in this case the lightweight Horizontal design, resulting in 82% lower pressure drop than the intermediate conventional design.

Furthermore, the findings of the secondary aim showed that, while the two absorber concept prototypes were successfully printed, multiple post-processing steps will be required for the part to be compliant and ready for use in the synchrotron. Areas of further research include techniques to decrease surface roughness, porosity and to achieve the required dimensional tolerances. Lastly, all the above findings can be applied to redesigning a shorter, full-size absorber with horizontal cooling channels on both sides of the BTS.

Finally, by leveraging the advantages of AM, a design focused on function rather than conventional manufacture has resulted in improved heat transfer, reduced pressure drop, part consolidation and a reduction in material waste, mass and improved potential lead times. The promising findings of the study suggest that further exploration and AM research in synchrotrons and particle accelerator applications is highly needed. This includes measuring the thermal conductivity of AM printed copper, which is expected to differ from OFHC copper; minimizing AM porosity; conducting transient thermal analyses; conducting experimental tests on the thermal performance; and pressure drop of the metal AM copper absorbers and quantifying the effects of AM surface roughness on both pressure drop and heat transfer.

## 6. Data availability

The data that support the findings of this study are openly available in eData, the STFC repository, including the relevant STL files and videos of pressure drop testing, both available in https://edata.stfc.ac.uk/handle/edata/972.

## Figures and Tables

**Table 1:** Material properties of copper and boundary conditions of the conventional (Conventional), Coil and Horizontal (Horizontal) design thermal simulation

Type	Boundary conditions	Value
OFHC copper properties	Thermal conductivity	391 W m^−1^ K^−1^
	Specific heat	385 J kg^−1^ K^−1^

DLS I04 beamline CPMU 17.6 mm period loading conditions	Beam power	6513 W
	Beam source distance	12.98 m
	Heat flux at BTS	Maximum: 14.8 W mm^−2^
	Ambient temperature	22°C
	Water cooling convection coefficient	Conventional and Coil: 12934 W m^−2^ °C^−1^
		Horizontal: 12211 W m^−2^ °C^−1^

**Table 2:** LPBF process parameters used for copper fabrication

Laser power	Scan speed	Hatch distance	Layer thickness
370 W	400 mm s^−1^	0.14 mm	30 µm

**Table 3:** Cylinder wall thickness measurement performed using a CMM ruby tip stylus

	1.5 mm CAD feature	1 mm CAD feature	0.5 mm CAD feature	0.25 mm CAD feature
Wall thickness (mm)	1.56	1.07	0.56	0.32
Standard deviation (3 meas.)	(0.00)	(0.00)	(0.00)	(0.00)

**Table 4:** Gyroid wall thickness measurement performed using a CMM camera

	0.96 mm CAD feature	0.64 mm CAD feature	0.32 mm CAD feature	0.16 mm CAD feature
Wall thickness (mm)	0.86	0.52	0.29	0.21
Standard deviation (3 meas.)	(0.02)	(0.01)	(0.03)	(0.02)

**Table 5:** Steady state thermal simulation results and comparison with intermediate conventional model

	Intermediate Conventional	Intermediate Coil	Intermediate Horizontal	Lightweight Horizontal
Maximum temperature	216.6°C	174.5°C	183.7°C	192.9°C
Difference from Conventional		20%	15%	11%

**Table 6:** Water properties used for convection coefficient Water properties at 22°C and 6 bar (gauge pressure)

Density (kg m^−3^)	Viscosity (mPa s)	Thermal conductivity (W m^−1^ K^−1^)	Specific heat (J kg^−1^ K^−1^)
998	1	0.60	4180

**Table 7:** Calculation of the convection coefficient at 3 m s^−1^

	Reynolds number	Prandtl	Nusselt	Convection coefficient (W m^2^ °C^−1^)
Horizontal	25108	6.62	162.2	12211
Conventional	18831		128.8	12934
Coil	18831		128.8	12934

**Table 8:** Fixed variable for each manifold and used fluid

	Water density (kg m^−3^)	Water viscosity (mPa s)	Flow rate (m^3^ s^−1^)	Pipe length (m)	ID (mm)
Horizontal	998	1	0.0001	0.42	8
Conventional				5.21	6
Coil				2.72	6

**Table 9:** Calculated flow velocity, Reynolds number and friction factor

	Flow velocity (m s^−1^)	Reynolds number	Friction factor
Horizontal	1.99	15887	0.027
Conventional	3.54	21182	0.026
Coil	3.54	21182	0.026

**Table 10:** Minor loss coefficients, *K*, for different pipe bends (Cengel & Cimbala, 2013 ▸)

Pipe bend	Loss coefficient, *K*
180° return bend	0.2
90° smooth bend	0.3

**Table 11:** Type and number of pipe bends in each manifold and the calculated equivalent length

	No. of 180° bends	Equivalent length (m)	No. of 90° bends	Equivalent length (m)
Horizontal	0	0	12	1.05
Conventional	19	0.89	0	0
Coil	0	0	79	5.57

**Table 12:** Calculated and experimentally measured major and minor pressure drop of each manifold design

Component	Major of manifold (bar)	Major of setup (bar)	Minor of manifold (bar)	Total (bar)	Measured (bar)
Horizontal	0.03	0.23	0.07	0.33	0.37
Conventional	1.39		0.24	1.85	–
Coil	0.72		1.48	2.43	3.68

## Appendix A Equations

### A1. Equations for calculating water cooling convection coefficient

Prandtl number equation (Cengel, 2004 ▸):

where Pr is the Prandtl number (dimensionless), μ is the dynamic viscosity of the fluid (mPa s), *C*_p_ is the specific heat capacity at constant pressure (J kg^−1^K^−1^) and *k* is the thermal conductivity of the fluid (W m^−1^ K^−1^).

Nusselt number for turbulent flow in tubes expressed by Dittus-Boelter equation (Cengel, 2004 ▸):

where Nu is the Nusselt number (dimensionless), Re is the Reynolds number (dimensionless), Pr is the Prandtl number (dimensionless) and *n* is a constant that is 0.4 for heating (fluid being heated) and 0.3 for cooling (fluid being cooled).

Reynolds number equation (Cengel, 2004 ▸):

where Re is the Reynolds number (dimensionless), ρ is the fluid density (kg m^−3^), *V*_avg_ is the average fluid velocity (m s^−1^),*D* is the hydraulic diameter of the tube (m) and μ is the dynamic viscosity of the fluid (mPa s).

Convective heat transfer coefficient equation expressed by Nusselt number (Cengel, 2004 ▸):

where *h* is the heat transfer coefficient (W m^−2^ K^−1^), Nu is the Nusselt number (dimensionless), *k* is the thermal conductivity of the fluid (W m^−1^ K^−1^) and *D* is the hydraulic diameter (m).

### A2. Equations for pressure drop calculation

Fluid velocity in tube equation (Çengel & Ghajar, 2015 ▸):

Pressure drop expressed by Darcy–Weisbach equation (Çengel & Ghajar, 2015 ▸):

where Δ*P* is the pressure drop (Pa), *f* is the Darcy–Weisbach friction factor (dimensionless), *L* is the length of the pipe (m), *D* is the diameter of the pipe (m), ρ is the fluid density (kg m^−3^) and *V*_avg_ is the average flow velocity (m s^−1^).

Friction factor for tubes with turbulent flow expressed by the Colebrook equation (Çengel & Ghajar, 2015 ▸):

where *f* is the Darcy–Weisbach friction factor (dimensionless), ε is the absolute roughness of the pipe (m), *D* is the diameter of the pipe (m) and Re is the Reynolds number (dimensionless).

Pressure drop minor losses expressed as equivalent length (Cengel & Cimbala, 2013 ▸):

where *L*_equiv_ is the equivalent length of the pipe (m), *K*_l_ is the minor loss coefficient (dimensionless), *D* is the diameter of the pipe (m) and *f* is the Darcy–Weisbach friction factor (dimensionless).

### A3. Conduction and convection heat transfer equations

Rate of conduction heat transfer expressed by Fourier’s law of heat conduction (Cengel, 2004 ▸):

where  is the rate of heat transfer by conduction (W), *k* is the thermal conductivity of the material (W m^−1^ K^−1^), *A* is the cross-sectional area perpendicular to the heat flow (m^2^) and  is the temperature gradient in the *x*-direction (K m^−1^).

Rate of convection heat transfer expressed by Newton’s law of cooling (Cengel, 2004 ▸):

where  is the rate of heat transfer by convection (W), *h* is the heat transfer coefficient (W m^−2^ K^−1^), *A*_s_ is the surface area through which convection heat transfer takes place (m^2^), *T*_s_ is the surface temperature (K) and *T*_∞_ is the temperature of the surrounding fluid (K).

## Appendix B Water cooling convection coefficient

Appendix *B*[App appb] provides the calculation of water heat transfer cooling convection coefficient used in thermal simulation of Section 2. The operating conditions of the cooling water at DLS are 22°C and a pressure of 6 bar, which result in a water density of 998 kg m^−3^ and a viscosity of 1 mPa s (see Table 6 ▸) (NIST, 2023 ▸). Considering a fixed velocity of 3 m s^−1^, the obtained Reynolds number [Appendix *A*[App appa], equation (3)] is Re > 10000, making the flow turbulent in all three designs. The Prandtl number [Appendix *A*[App appa], equation (1)] considering a water thermal conductivity of 0.60 W mK^−1^ and a water specific heat of 4180 J kg^−1^ K^−1^ results in a Prandtl value of 6.62 (see Table 7 ▸). Considering our case of heated flow (hot wall, cold fluid), the *n* number was set to 0.4 (Seibold *et al.*, 2022 ▸), the Nusselt number was calculated using the Dittus-Boelter equation [Appendix *A*[App appa] equation (2)] and the convective coefficient was calculated using equation (4) of Appendix *A*[App appa], with results shown in Table 7 ▸.

## Appendix C Pressure drop calculation

Appendix *C*[App appc] provides the calculation of the pressure drop of the conventional, Horizontal and Coil designs. The fixed variables in this calculation are water properties at 20°C, the pipe length and ID of each setup and a flow rate of 6 L min^−1^ (see Table 8 ▸) making the flow turbulent in all three designs (see Table 9 ▸). Major losses covered the manifold and experiment setup pipe lengths, and uses the Darcy–Weisbach and Cole­brook equations [Appendix *A*[App appa], equations (5) and (6)]. Minor losses covered the cooling pipe geometry bend losses, which were estimated using the equivalent length equation [Appendix *A*[App appa], equation (7)] and minor loss coefficients seen in Table 10 ▸. The recommended absolute roughness value (ε) for commercial plastic pipes is 0 (smooth) (Cengel & Cimbala, 2013 ▸) and, in this case, selected to represent the 3D printed resin pipes. The conventional design is composed of 180° bends, and both the Horizontal and Coil designs are assumed to be composed of 90° bends (see Table 11 ▸), of which the loss coefficients can be found in Table 10 ▸. The ‘Major of Setup’ seen in Table 12 ▸ was estimated from the test rig pipe lengths measuring 3.3 m and an ID of 8 mm. Minor losses caused by the test rig setup fittings were not calculated.
